# Self-Healing Behavior of Medium-Temperature Thermoresponsive Polyurethane-Modified Asphalt: Multiscale Structure–Property Relationships

**DOI:** 10.3390/polym18212711

**Published:** 2026-11-01

**Authors:** Kenan Li, Dengke Yang, Lang Liu, Mengbo Zhu, Zhizhen Liu, Tao Ai

**Affiliations:** 1 School of Civil and Architectural Engineering, Xi’an University of Science and Technology, Xi’an 710054, China; 2 College of Energy and Mining Engineering, Xi’an University of Science and Technology, Xi’an 710054, Chinambzhu@xust.edu.cn (M.Z.); zzliu0217@163.com (Z.L.); 3 School of Materials Science and Engineering, Chang’an University, Xi’an 710064, China; aitao@chd.edu.cn

**Keywords:** thermoresponsive polyurethane modified asphalt, dynamic disulfide bond, healing performance, structure–property relationship, molecular dynamics

## Abstract

Asphalt surface layers are prone to deterioration under long-term vehicle loads and environmental factors, making active healing binders essential for pavement durability. This study designed a polyurethane-modified asphalt (PUA) with dynamic disulfide bonds that can be thermally activated at medium temperatures (40–60 °C). Rheological tests, adhesion tests, and molecular simulations revealed the relationship between chemical composition, molecular structure, and healing performance from molecular to macroscopic scales. Results showed that asphalt cohesion and asphalt–aggregate bond strength were key intrinsic factors. Compared with the disulfide-free PUA, the DSDDP-containing formulations exhibited higher fatigue-healing indices and modest improvements in adhesion-healing indices under the tested conditions. For PUA with a DSDDP molar fraction of 10% in composite chain extender, the fitted healing curve predicted that the complex-modulus-based healing index (HI_1_) would reach 0.9 after approximately 680 s at 50 °C. At construction temperatures (>135 °C), dynamic exchange reduced network density and improved workability. Molecular dynamics simulations demonstrated that diffusion and movement accelerated with increasing initial spacing and rising temperature. S-S bonds reduced intermolecular forces via topological rearrangement, accelerating migration toward cracks and forming a denser structure after healing. This thermoresponsive PUA mitigates crack formation and expansion, enabling sustainable pavement applications.

## 1. Introduction

The surface layer of asphalt pavement is prone to fatigue cracking and other diseases during long-term service [1,2]. As the number of load cycles increases, residual deformation gradually accumulates, leading to the formation of fatigue cracks in pavement, which adversely affects its short-term performance and durability [3,4]. The durability of asphalt encompasses weather resistance against prolonged climatic exposure and healing capacity under fatigue states [5,6]. Enhancing the healing performance of asphalt facilitates rapid self-repair after the appearance of micro-cracks and minor damage [7]. This enables asphalt pavements to withstand heavier and more frequent traffic loads, thereby improving driving safety and reducing the overall maintenance frequency and cost for road infrastructure [8,9].

The healing performance of asphalt refers to the ability to partially restore its original properties through physical and chemical changes after sustaining damage [10]. This self-healing capacity is mainly explained by surface energy theory and molecular diffusion theory [11]. When asphalt develops cracks due to prolonged external forces, its surface energy increases, rendering asphalt molecules unstable and endowing an inherent driving force to heal the cracks [12]. The asphalt on both sides of the crack wets and adheres to each other, migrating towards the center [13,14]. According to the principle of molecular diffusion, asphalt molecules tend to move from areas of higher concentration to lower concentration, achieving uniform distribution [15]. Consequently, they diffuse towards cracks and rearrange to gradually fill the cracks [16]. It is evident that surface energy motivates asphalt molecules on both sides of cracks to approach, gradually achieving dynamic equilibrium during molecular diffusion [17].

The healing capacity of asphalt is jointly influenced by multiple internal and external factors and is closely associated with its adhesion capacity [18]. The primary internal factors include the composition, chemical properties and microstructure of asphalt [19]. In general, adding polymers to the base asphalt significantly enhances its viscoelasticity and adhesion to aggregates, while also improving stress relaxation ability and low-temperature flexibility [20]. This enables modified asphalt to disperse stress and return to its original state more rapidly after the external forces cease, thereby alleviating the formation of cracks [21]. The external factors affecting healing performance mainly include the healing temperature, intermission, loading condition and stress level [22]. At elevated temperatures, asphalt flows more rapidly, and the intermolecular forces are enhanced, facilitating the recombination of molecules at cracks [23]. As the stress level increases, the degree of fatigue damage to asphalt intensifies, hindering its rapid healing and performance recovery. Dynamic and fatigue loading can induce microcracks inside the material, while high-amplitude and high-frequency loading may cause severe damage to asphalt [24]. Therefore, heavy traffic roads impose higher demands on the healing properties of asphalt binder.

The structure–property relationship of asphalt refers to the correlation between chemical composition, molecular structure and arrangement, microstructure, and its macroscopic performance [25]. Currently, the primary technologies for enhancing the healing performance of asphalt include active and passive methods [26,27]. The former involves modifying the composition and structure of asphalt, such as micro-capsule technology, nano-modification technology, and polymer modification technology [28]. The micro-capsule leverages contact reactions to enhance healing efficiency, but it incurs high production costs and is disposable, with poor dispersion uniformity in the mixture [29]. Nano-modification technology exhibits low environmental dependence, but it also faces challenges of high cost and great technical complexity [30]. Polymer modification technology is suitable for various climatic and environmental conditions, but the repair effectiveness varies significantly, and the construction process is complicated [31].

Passive healing technology refers to introducing external fields into asphalt mixtures to generate greater energy. The main methods include electromagnetic induction heating and microwave heating under thermal initiation conditions or forming reversible chemical bonds in asphalt, such as photo-repair [32]. The electromagnetic induction heating method involves adding conductive fibers or fillers to form a closed circuit around cracks, thereby melting the asphalt and repairing the crack. This method offers the advantages of being fast and efficient, but it requires specialized supporting equipment and technology. In contrast, the microwave heating method is highly efficient and rapid, which is suitable for large-scale pavement repair, but it requires high equipment costs and presents significant construction challenges [33]. In addition, the photo-repair method involves adding repair agents with a photocatalytic effect to asphalt, which breaks the bonds under ultraviolet light and forms free radicals that can be recombined [34]. This method can be repeated and has simple triggering conditions, but it is not suitable for repairing deep cracks on the pavement surface.

It is evident that the current active healing technologies have disadvantages such as high cost, complex processes, and significant variation in healing efficiency. Meanwhile, passive healing technologies remain immature, making it difficult to apply in practical engineering projects. To identify a technology capable of long-term stability and active repair rapidly after crack formation, the self-healing system of PU materials from polymer chemistry was adopted. The intermolecular forces of PU gradually weaken during melting and partially recover upon cooling. This reversible chemical change demonstrates that PU possesses certain self-healing abilities [35]. However, the strength of hydrogen bonds is much weaker than that of covalent bonds, which severely limits the healing performance of PU [36]. In contrast, introducing stronger and more flexible dynamic covalent bonds into the PU macromolecular structure is conducive to the performance recovery and durability enhancement of PU [37]. The synthesized PU material can actively repair the existing damage under the stimulation of external conditions (such as light exposure, heating and mechanical force), thereby restoring its original properties [38].

At present, reversible covalent bond systems are mainly divided into intrinsic self-healing systems based on thermal initiation and photo initiation [39,40,41]. The former includes self-healing based on reversible Diels-Alder (DA) reactions and reversible dynamic covalent bonds [42]. The DA reaction exhibits high regioselectivity and stereoselectivity, but the reverse reaction of DA bonds only occurs when PU materials are heated above 100 °C [43]. The elevated healing temperature increases the technological complexity and may cause thermal deformation or damage around the repair area during the healing process [44]. In contrast, incorporating repeatedly breakable and connectable disulfide (S-S) covalent bonds into PU helps to improve its healing efficiency [45]. This is because S-S bonds have lower bond energy (240 kJ/mol), require relatively simple activation conditions (medium temperature 40–60 °C or ultraviolet irradiation), and enable PU materials to exhibit highly efficient ambient-temperature self-healing performance without catalysts during reaction [46,47,48]. Due to the broader application scope of this active healing system, it has been initially introduced into PUA to improve its durability [49]. However, the relationship between time-dependent viscosity changes at construction temperatures and healing recovery at moderate temperatures remains insufficiently characterized, particularly with respect to chain-extender composition [50]. The relatively low S–S bond energy provides a thermodynamic basis for exchange, but the activation temperature is governed by exchange kinetics, chain mobility, and the local chemical environment [51,52]. In this study, 40–60 °C was selected because it matches pavement service temperatures and was experimentally supported by rheological and healing tests, rather than being inferred from bond energy alone.

**Figure 1:**
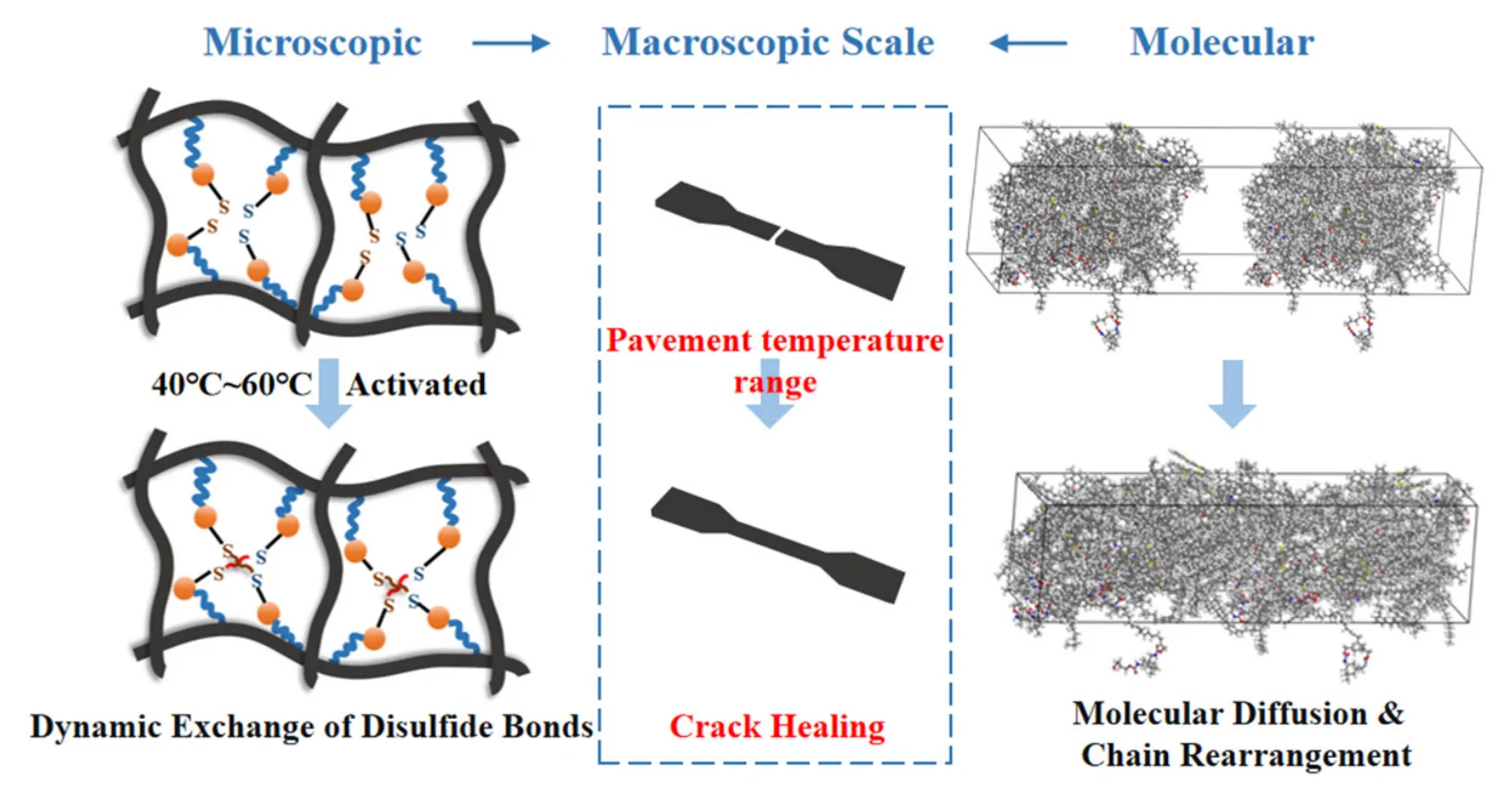
Active performance recovery effects of S-S bonds.

At moderate temperatures, increased molecular mobility can promote interfacial contact and healing recovery, but it may also increase susceptibility to deformation under loading [53]. The performance of disulfide-containing PUA therefore depends on the balance between molecular mobility and structural stability [54,55]. To expand the application potential of active healing PUA in long-life asphalt pavement and maintain the balance between healing efficiency and structural stability, this study investigated the adhesion-healing and fatigue-healing behavior of thermoresponsive PUA based on dynamic S-S bond systems under different intermissions. In this way, the workability enhancement and active performance recovery effects of S-S bonds on PUA were elucidated (as shown in Figure 1). Subsequently, the healing process of PUA after the formation of microcracks was simulated at the molecular scale, and the diffusion and motion characteristics of its components were analyzed. The objective of this study is to design a medium-temperature thermoresponsive PUA based on dynamic disulfide bonds, evaluate its fatigue-healing and adhesion-healing performance, and establish a multiscale structure–property relationship from molecular to macroscopic scales.

## 2. Materials and Methods

### 2.1. Raw Materials and Preparation Process

This study used thermoplastic polyolefin-based PUA, which was composed of 70# base asphalt and PU. The soft segment material of PU is hydroxyl-terminated polybutadiene (HTPB, 99%, Shenzhen Hongyuan Chemical New Materials Technology Co., Ltd., Shenzhen, China), while the hard segment primarily consists of isophorone diisocyanate (IPDI, 99.5%, Alpha Chemical Co., Ltd., Tianjin, China) and composite chain extenders including 1,4-butylene glycol (BDO, 99.5%, Xilong Chemical Co., Ltd., Shantou, China) and 4,4′-disulfanediyldiphenol (DSDDP, 98%, Alpha Chemical Co., Ltd.). Specific parameters of raw materials and 70# base asphalt can be viewed in Supplementary File Section S1 [56]. It is worth noting that DSDDP contains disulfide (S-S) covalent bonds that are capable of conducting effective dynamic exchange and topological rearrangement at medium temperatures (40–60 °C). This property contributes to enhancing the flowability of synthesized PU at high temperatures and its self-healing capacity under pavement surface temperature ranges.

**Figure 2:**
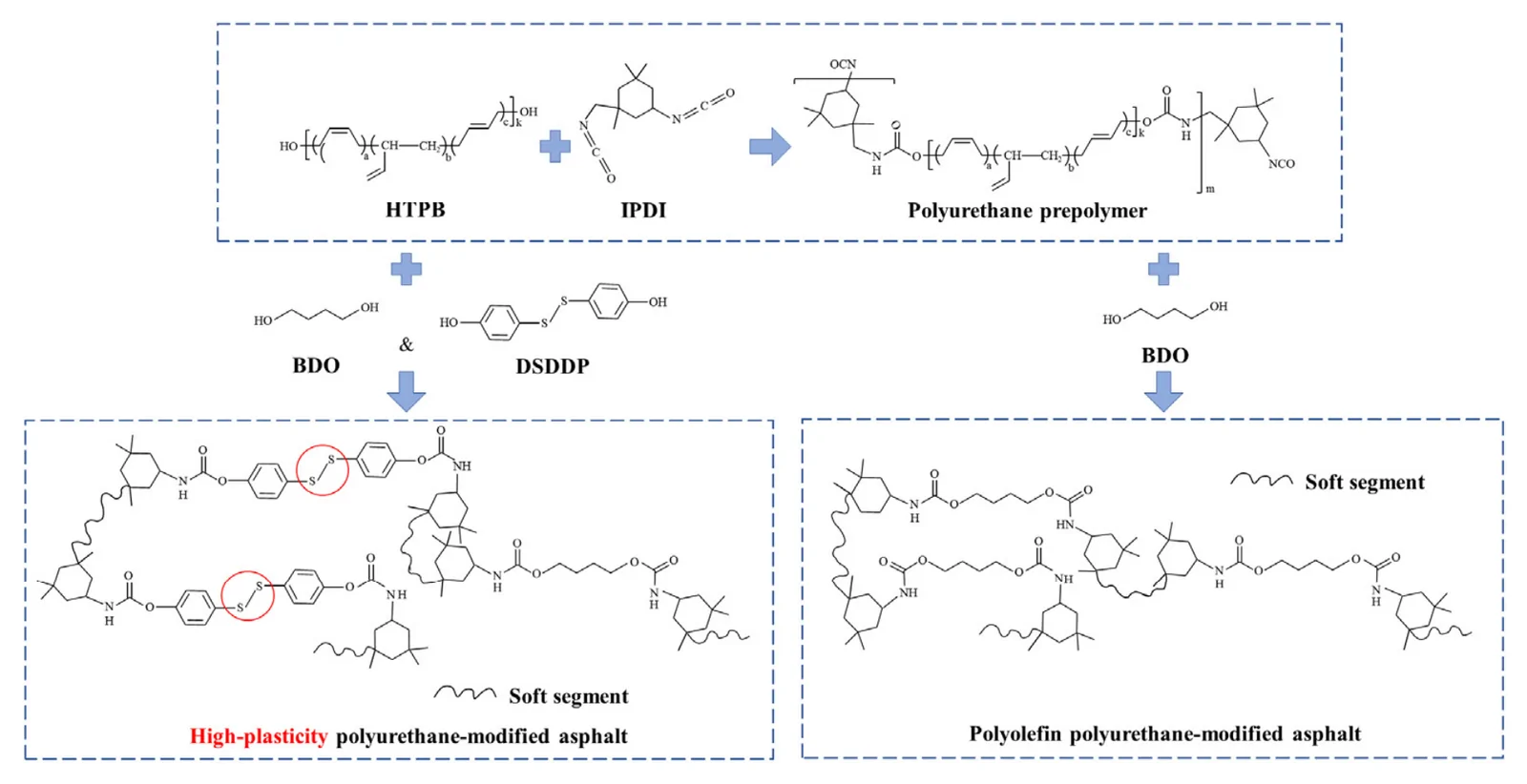
Chemical reactions during the preparation of PUA.

In terms of the preparation process, the prepolymer method was selected for its great controllability of product performance [57]. The specific process was as follows: Firstly, IPDI and HTPB were reacted at 80 °C under vacuum nitrogen conditions for 2.5 h to prepare the PU prepolymer [58]. It was then added to the preheated 70^#^ asphalt and sheared at 800 rpm for 1 h at 145 °C [59]. Subsequently, the chain extenders were added, and shearing was continued for 30 min. Finally, the product was maintained in an oven at 150 °C for 8 h. The main chemical reactions are illustrated in Figure 2, involving the nucleophilic addition reaction between the -NCO groups of IPDI and -OH groups at both ends of HTPB, as well as the subsequent consumption of excess -NCO groups in the PU prepolymer through the active hydrogen at both ends of the small-molecule chain extender. Linear polymer chains of varying lengths were formed.

It has been determined in our previous studies that when the PU content was 20%, while maintaining the ratio of n(IPDI): n(HTPB) at 3:1 and the R value (-NCO/-OH) at 1.0, the high-temperature rheological properties of PUA containing dynamic S-S bonds approached those of SBS-modified asphalt, while exhibiting superior low-temperature flexibility [60]. Based on preliminary trials and production cost considerations, the molar fraction of DSDDP in the composite chain extender was limited to 40%. Formulations containing 10% and 40% DSDDP molar fraction were selected to examine the effect of chain-extender composition on the flow and healing properties of PUA. Accordingly, three PUA formulations were prepared with BDO:DSDDP molar ratios of 6:4, 9:1, and 10:0, corresponding to DSDDP molar fractions of 40%, 10%, and 0% in the chain extender component, respectively. The complete formulation table is presented in Table 1. They were designated as 20%HTPUA6:4, 20%HTPUA_9:1_ and 20%HTPUA_BDO_. Additionally, high-viscosity SBS-modified asphalt commonly used in pavement construction was selected for comparison. The SBS modifier employed a star-shaped structure with an addition rate of approximately 8%.

### 2.2. Fourier Transform Infrared Spectroscopy Tests

To verify the reactions that occurred during the synthesis of polyurethane-modified asphalt, Fourier transform infrared spectroscopy (FTIR) (Bruker Optics, Billerica, MA, USA) tests were conducted on 3 groups of prepared PUA- and SBS-modified asphalt. The attenuated total reflection (ATR) method was used, with each sample scanned 32 times over a wavenumber range of 500 cm^−1^ to 4000 cm^−1^.

### 2.3. Plasticity and Flowability Tests

The molecular motion of asphalt is restricted below 0 °C, and its plastic behavior manifests as ductile fracture caused by stress concentration. Therefore, according to the T 0604 and T 0605 methods specified in standard JTG E20-2011 [61], the penetration at 25 °C and ductility at 5 °C of different PUA were tested, respectively. Asphalt exhibits viscoelastic fluidity at elevated temperatures (above 60 °C). The temperature sensitivity of PUA was evaluated by the softening point in method T 0606 and the Brookfield viscosity in method T 0625. The viscosity test was conducted by using a No. 28 rotor at a rotational speed of 20 r/min. Test temperatures were set at 115 °C, 135 °C, and 155 °C to simulate the temperature range during construction. Each test was repeated three times, and results are reported as mean standard deviation. Samples were equilibrated at the test temperature for 15 min before measurement. Viscosity was considered stable when three consecutive readings varied by less than 1% or 0.05 Pa·s. The high-temperature flowability of different asphalt was investigated by analyzing the trend of viscosity over time. After that, the sample was left undisturbed for 30 min, and the viscosity test was conducted again to assess its viscoelastic recovery capability.

### 2.4. Healing Performance Tests

#### 2.4.1. Fatigue–Healing–Fatigue Test

Pavement binding materials develop fatigue damage and cracks under prolonged traffic loads. The recovery capacity of asphalt after fatigue can characterize its damage accumulation and healing behavior. The fatigue–healing–fatigue test was conducted through a TA Discovery HR–2 dynamic shear rheometer (DSR). Specimen slip was checked by monitoring the torque–strain relationship. The specific process procedure involved first performing a time-scanning test on different asphalt under a stress condition of 0.1 MPa, with a loading frequency of 10 Hz. The initial complex modulus ($G_{0}^{*}$) of the samples was recorded. Loading was then paused when the complex modulus decreased to 60% of $G_{0}^{*}$, followed by a designated intermission for asphalt healing. Subsequent loading resumed until the damage degree reached 40% again. Tests were repeated three times. The 60% and 40% modulus thresholds were selected based on the transition from stable fatigue development to accelerated failure. The above test adopted 8 mm parallel plates with a 2 mm loading gap. The healing indexes were determined by the change rate in complex modulus of asphalt before and after healing (*HI*_1_) and the ratio of cumulative dissipated energy (*HI*_2_), which are calculated according to Equations (1) to (4), respectively. The absolute fatigue endurance was quantified as the loading time and the number of cycles required for G^*^ to decrease to 60% of $G_{0}^{*}$ during the first loading stage, and to reach the 40% damage threshold after healing.

(1) $${HI}_{1}=\frac{G_{a}^{*}-G_{b}^{*}}{G_{0}^{*}-G_{b}^{*}}\cdot \frac{t_{2}}{t_{1}}$$

*HI*_2_ = *W*_a_/*W*_b_ (2)

(3) $$W=\Sigma_{i=1}^{n}W_{i}$$

*W*_i_ = π*σε*_i_sin*δ*_i_ (4)

where $G_{a}^{*}$ denotes the initial complex modulus after healing (kPa); $G_{b}^{*}$ is the complex modulus before healing (kPa); *t*_1_ and *t*_2_ represent the loading times before and after healing, respectively (s); *W*_b_ and *W*_a_ are the cumulative dissipated energy before and after healing, respectively (mJ/m^3^); *W*_i_ is the dissipated energy within a certain loading cycle (mJ/m^3^); *σ* and *ε*_i_ are the loading stress (MPa) and dimensionless strain; *δ*_i_ is the phase angle (°).

#### 2.4.2. Adhesion–Healing–Adhesion Test

The adhesion capacity of asphalt is the foundation for the healing process. To further investigate the influence of bond strength on asphalt healing performance, the Binder Bond Strength (BBS) test was first conducted using a Positest AT-A tester (DeFelsko, Ogdensburg, NY, USA) in accordance with the AASHTO TP 91-15 standard [62]. The size of the limestone substrate was 100 mm × 100 mm × 20 mm, with a loading rate of 690 kPa/s, yielding the initial pull-off tensile strength (*POTS*_Initial_). After the first pull-off test, the stub was immediately repositioned using an alignment jig. Contact pressure, alignment, and binder film thickness were controlled by the stub weight, the jig, and the coating amount. The specimens were cured at 60 °C for 12 h. After cooling to room temperature, the specimens were subjected to a second BBS test at the same loading rate to measure the POTS after healing (*POTS*_Healing_). Each PUA sample underwent 3 repeated tests, with the average value recorded as the result. The adhesion-healing efficiency of PUA is evaluated according to the healing index (*HI*_3_) in Equation (5). Furthermore, to examine the ability of asphalt to maintain structural integrity under long-term water erosion, another set of parallel specimens after undergoing the BBS test were immersed in a water bath at 60 °C for 12 h, and then kept at 25 °C for 2 h after removal. Subsequently, their *POTS*_Healing_ was tested to assess the water immersion adhesion-healing performance.

*HI*_3_ = *POTS*_Healing_/*POTS*_Initial_ (5)

### 2.5. Construction of PUA Molecular Healing Models

Molecular dynamics (MD) simulation can reveal the molecular motion states and interaction changes in asphalt during the healing process from a nanoscale perspective, thereby reflecting the rearrangement and diffusion of molecular chains at microcracks. Firstly, a 12-molecule model of asphalt was constructed based on the improved AAA-1 four-component asphalt model, and 2 PUA molecules were established, which can be viewed in Supplementary File Section S2 [63]. The specific construction process is as follows: By using the “Build Polymers” module in Materials Studio 8.0 (MS) software, PU modifier models with chain extenders entirely composed of BDO or DSDDP were established in ratios of n(HTPB): n(IPDI): n(BDO) = 1:3:2 and n(HTPB): n(IPDI): n(DSDDP) = 1:3:2, respectively. These two models represent the lower and upper bounds of S–S content, respectively, and were used to isolate the intrinsic effect of the S–S-containing chain extender on molecular diffusion, interfacial interaction, and cohesive energy during healing. It was calculated that inserting 4 PU molecular chains into the asphalt molecular model resulted in a PU content of approximately 20% of asphalt, which was close to the PUA prepared in the laboratory. The “Amorphous Cell Calculation” module was then used to assemble the molecular models of base asphalt and PU modifiers, and the PUA models were named HTPUA_BDO_ and HTPUA_DSDDP_, respectively.

**Figure 3:**
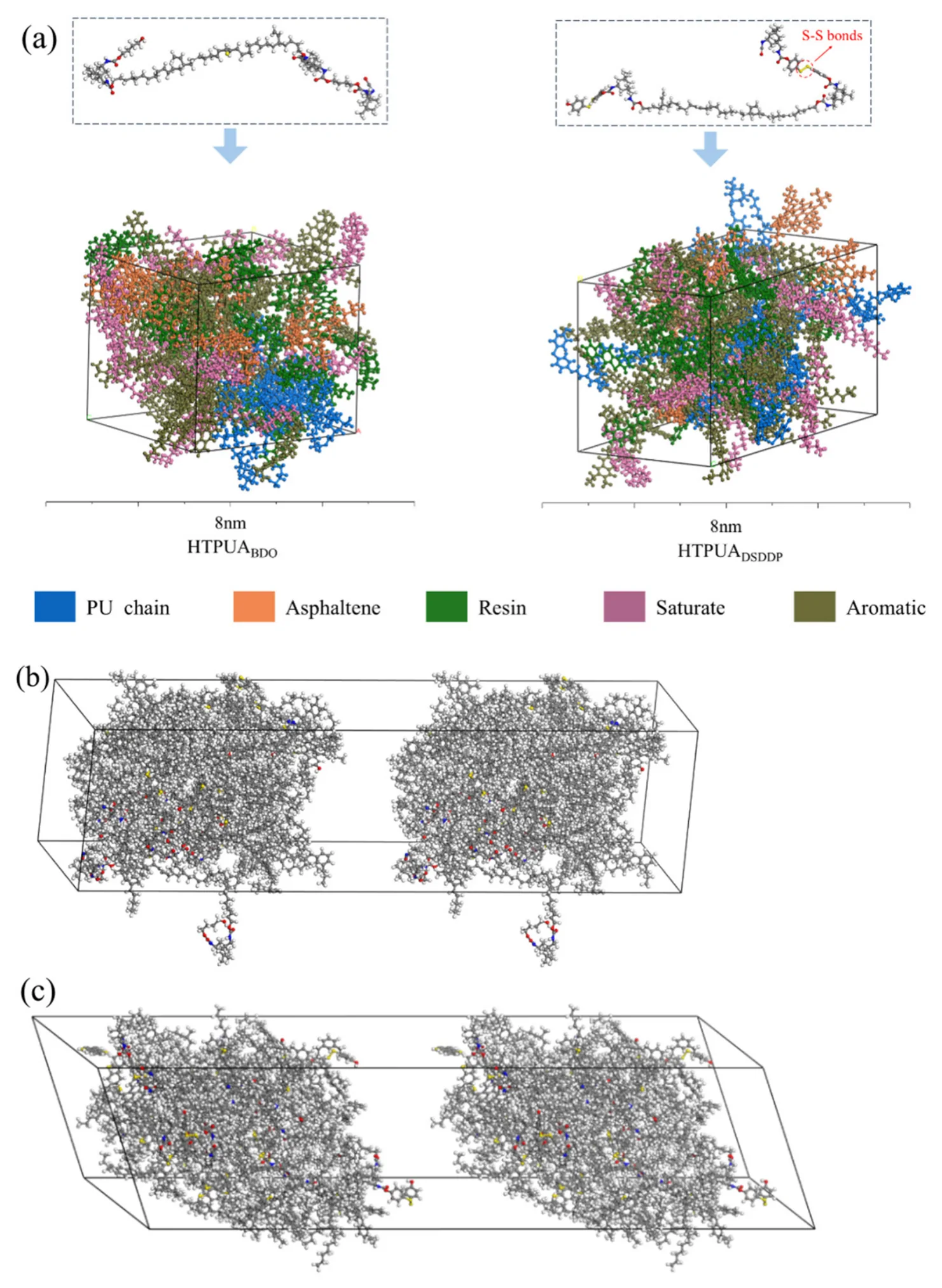
(**a**) PUA molecular models, initial PUA healing models of (**b**) HTPUA_BDO_/10 Å and (**c**) HTPUA_DSDDP_/10 Å.

COMPASS is a first-principles-based force field that enables accurate prediction of structural, conformational, vibrational, and thermophysical properties for a wide range of molecules and condensed phases. COMPASS II is specifically parameterized to yield reliable interaction properties between organic substances. The selection of COMPASS II was primarily based on its proven accuracy and adaptability for asphalt-related molecular systems compared with different force fields (UFF and Dreiding). Subsequently, the “Forcite” module was adopted to conduct energy optimization and 40,000 steps of geometric optimization on the constructed molecular models, thereby identifying the lowest energy molecular configuration. The models were subjected to annealing optimization for 5 cycles with a total of 150 ps steps and within the temperature range of 25 °C to 325 °C under the NVT ensemble, thus eliminating local high-energy points. Finally, a 500 ps MD simulation was carried out under the NPT ensemble to ensure thorough mixing and stabilization of molecules within the system. The final PUA molecular models are shown in Figure 3a.

To simulate the microcracks generated during service at the molecular scale, 2 identical layers of HTPUA_BDO_ or HTPUA_DSDDP_ that underwent 150 ps of MD simulation were imported through the “Build Layers” function in MS software (Materials Studio 8.0). Vacuum layers of 10 Å (1 nm), 30 Å and 50 Å were constructed, respectively, between the 2 asphalt molecular layers to represent different crack widths, which macroscopically correspond to different degrees of asphalt damage. Finally, 6 groups of initial PUA healing models were constructed, as partially illustrated in Figure 3b,c. To simulate the changes that occurred in asphalt models with different gaps during the healing process, the system was first subjected to energy optimization and 2000 steps of geometric optimization. Subsequently, 500 ps of MD simulations were conducted under the NPT ensemble at simulated pavement temperatures of 25 °C, 45 °C, and 65 °C.

## 3. Results

### 3.1. Chemical Structure Characterization

**Figure 4:**
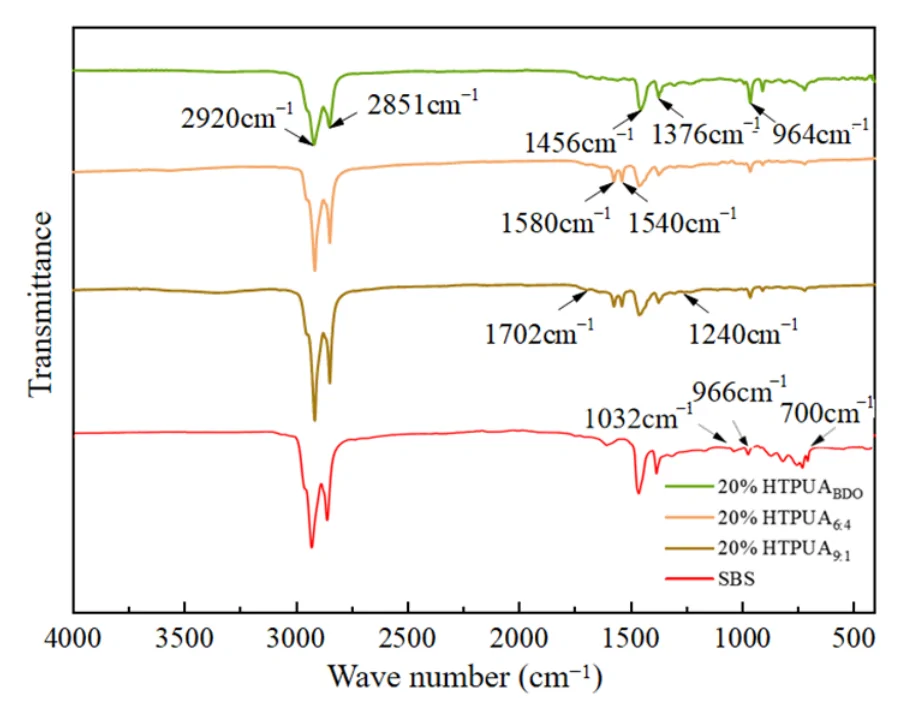
FTIR spectra of PUA- and SBS-modified asphalt.

FTIR spectra of different PUA- and SBS-modified asphalt are shown in Figure 4.

Figure 4 demonstrates that after the incorporation of different types of PU modifiers into asphalt, the stretching vibrations of C=O bonds and C-O bonds on the amide group of PUA could be observed at 1702 cm^−1^ and 1240 cm^−1^, respectively. The absorption peak at 964 cm^−1^ was attributed to the carbon-carbon double (C=C) bond in the soft segment HTPB, while the small absorption peak at 1540 cm^−1^ corresponded to the C=C skeletal vibration of the benzene ring in the DSDDP structure. All these indicated that the components of PU had completely reacted and were uniformly dispersed in the asphalt medium and were chemically modified with asphalt. In comparison, the absorption peaks of SBS-modified asphalt at 966 cm^−1^ and 700 cm^−1^ corresponded to the stretching vibration of C=C and the bending vibration of C-H, while the peak at 1032 cm^−1^ represented the S=O bonds in the sulfoxide group.

### 3.2. Flow and Deformation Characteristics

#### 3.2.1. Basic Properties and Viscosity-Temperature Sensitivity

**Figure 5:**
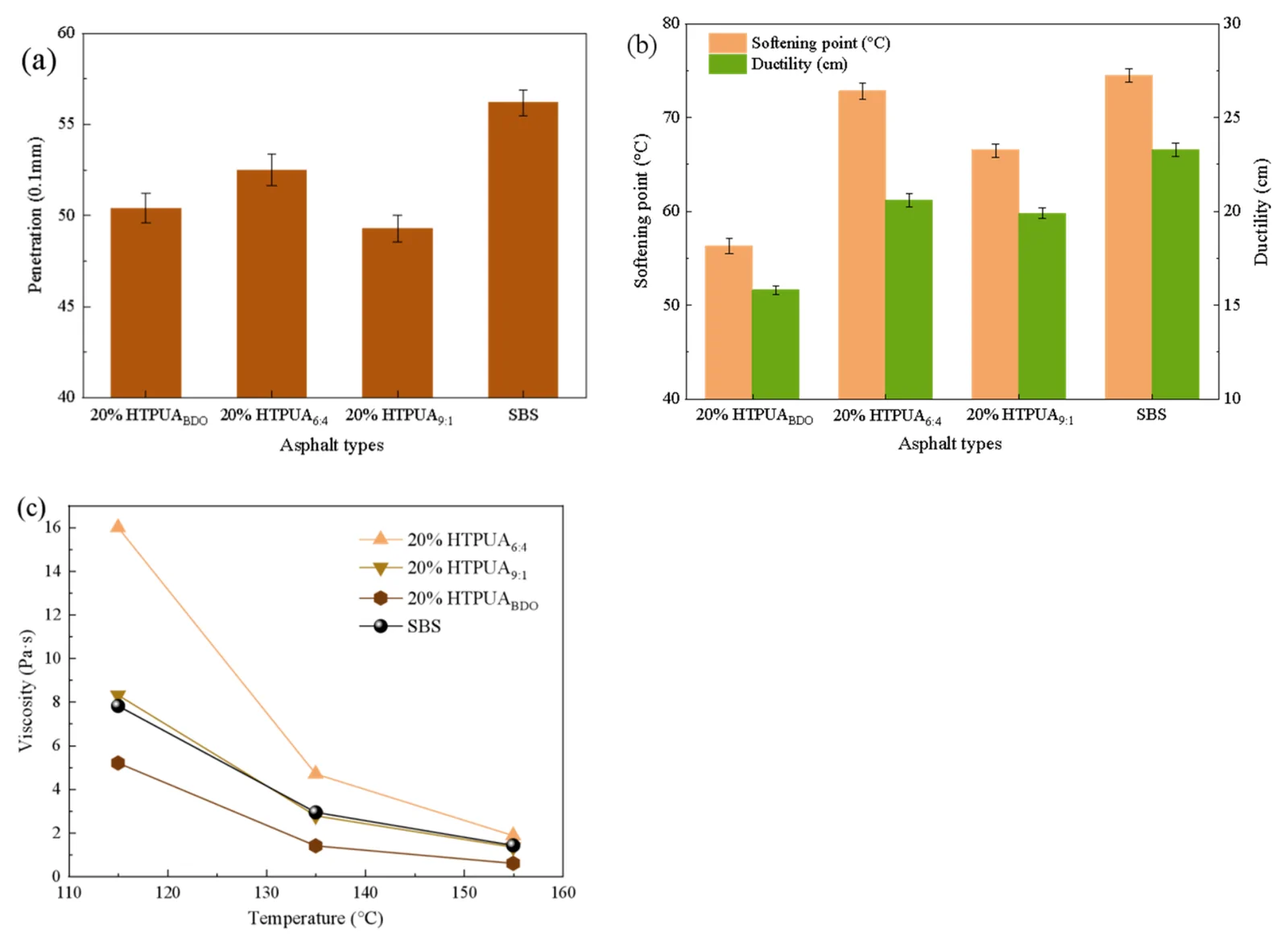
Basic properties of modified asphalt: (**a**) Penetration, (**b**) softening point and ductility and (**c**) viscosity.

The 25 °C penetration, 5 °C ductility, and softening point of SBS-modified asphalt and 3 groups of PUA are presented in Figure 5a,b, and their rotational viscosity at different temperatures is shown in Figure 5c.

As illustrated in Figure 5a,b, the introduction of DSDDP as part of the chain extenders significantly increased the penetration, softening point and ductility of PUA. This demonstrated that the incorporation of S-S bonds was effective in enhancing the resistance of PUA to high-temperature flow deformation and low-temperature plastic deformation. At a DSDDP content of 40%, the penetration, softening point and ductility of 20%HTPUA_6:4_ exceeded 52 (0.1 mm), 70 °C and 20 cm, respectively. Its basic physical properties approached SBS-modified asphalt, meeting the technical specifications for high-viscosity asphalt in pavement construction.

Figure 5c indicated that the viscosity of PUA containing DSDDP was substantially increased. The viscosity of 20%HTPUA_9:1_ was essentially comparable to SBS-modified asphalt, which exceeded 3 Pa·s at 135 °C. Moreover, the effect of viscosity enhancement became more significant with the increase in S-S bond content. This suggested that the addition of S-S bonds facilitated the formation of a denser crosslinked structure of PU in asphalt, thereby improving its cohesive force. Consequently, controlling the appropriate S-S bond content could improve the hardness and intensity of PU, ultimately strengthening the resistance of modified asphalt to shear stress.

**Figure 6:**
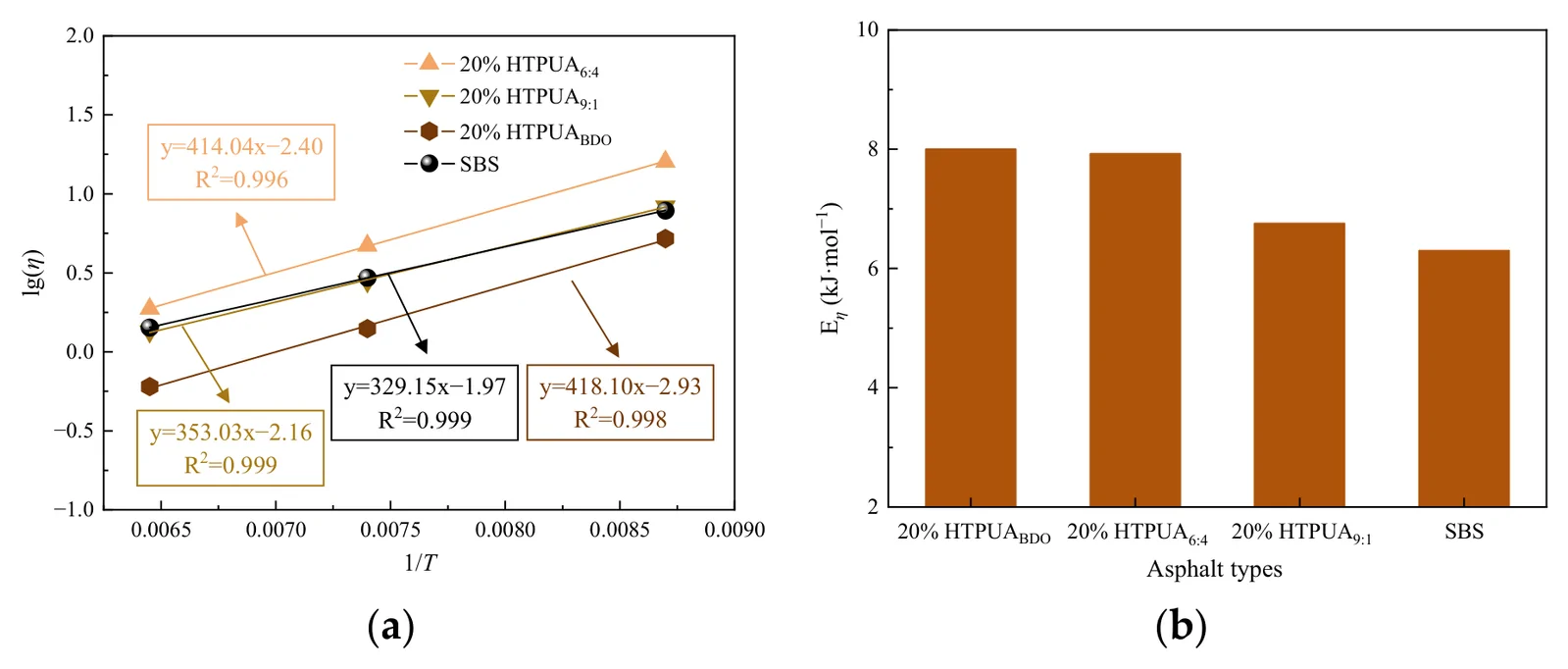
(**a**) Arrhenius fitting curves and (**b**) *E_η_* of modified asphalt.

The correlation between viscosity and temperature (*T*) directly affects the workability of asphalt during construction. The Arrhenius equation and its transformation reflect the temperature dependence of chemical reaction rate constants, as detailed in Equations (6) to (7) [64]. The visco-flow activation energy (*E_η_*) quantifies the flowability of asphalt, thereby evaluating its temperature sensitivity. Higher temperature sensitivity of asphalt indicates stronger flowability at elevated temperatures. The Arrhenius fitting curves of different asphalt and the further calculated *E_η_* are presented in Figure 6.

(6) $$\eta (T)=Ke^{\frac{E_{\eta }}{RT}}$$

(7) $$\mathrm{lg}\left(\eta \left(T\right)\right)=\mathrm{lg}\left(K\right)+\frac{E_{\eta }}{2.303\times \mathrm{RT}}$$

where *K* is the material constant; *R* is the molar gas constant, *R* = 8.314 J(mol·K).

Figure 6a indicates a significant linear relationship between lg(*η*(*T*)) and 1/*T* for different asphalts. A lower *E_η_* indicates weaker temperature dependence. The PU network should be described as a dynamic, reversible physical–covalent network rather than a permanently covalently crosslinked network. It is calculated that after adding 10% DSDDP, the *E_η_* of PUA was greatly reduced, and its temperature sensitivity was slightly higher than that of SBS-modified asphalt. This suggested that the introduction of S-S bonds enhanced the stiffness of PU molecular chains, enabling PUA to maintain stability under large temperature differentials. The flow and deformation behavior was effectively suppressed at high temperatures, and asphalt was thus less prone to cracking. In contrast, *E_η_* increased when the DSDDP content rose to 40%. This is attributed to the increased content of exchangeable S-S bonds in the system, which increased asphalt fluidity through constant bond breaking and recombination.

#### 3.2.2. Thermoresponsive Flow and Deformation Behavior

**Figure 7:**
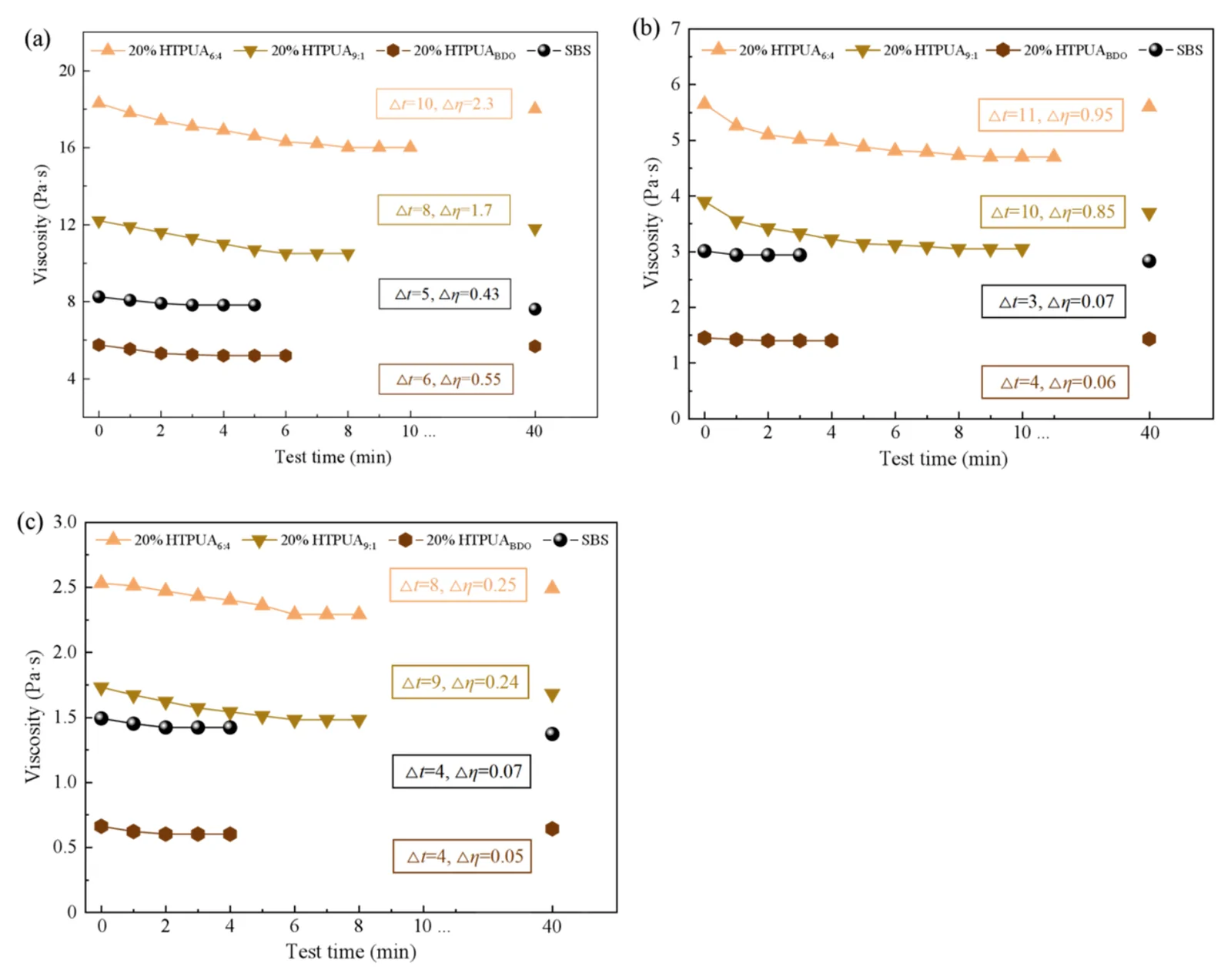
Viscosity changes with test time at (**a**) 115 °C, (**b**) 135 °C and (**c**) 155 °C of modified asphalt.

The flow and deformation capacity of asphalt refers to its ability to generate irreversible plastic deformation under external forces while maintaining some of its properties after the removal of forces. The enhancement of its flowability within the construction temperature range is beneficial for the workability of asphalt mixture. The changes in viscosity of PUA- and SBS-modified asphalt with test time at different temperatures are shown in Figure 7. The thermoresponsive flowability of asphalt was evaluated by defining the time required to reach a stable viscosity state as Δ*t* (min) and the variation in viscosity during this period as Δ*η* (Pa·s), where Δ*η* = Δ*η*_Initial_ − Δ*η*_Stable_.

It can be seen from Figure 7 that at a certain rotational speed, the viscosity of modified asphalt first decreased and then gradually stabilized as the duration of external force increased. This is due to the constant shear force on asphalt starting from a static state at high temperatures, manifesting as an internal friction force from inner to outer layers, which partially disrupted molecular structures. The viscosity gradually increased and recovered to its original state during the intermission. From a microscopic perspective, the polar dispersed phase PU and the dispersed medium asphalt molecules returned to a low-energy state through stress relaxation and rearranged to achieve electrostatic equilibrium, which reflected the thixotropy of PUA. Although a larger viscosity drop and longer stabilization time indicate stronger structural rearrangement, workability should be judged together with the absolute stabilized viscosity. At 135 °C, 20%HTPUA_9:1_ showed a stabilized viscosity comparable to SBS, whereas higher DSDDP contents may increase mixing and compaction difficulty.

The Δ*t* of SBS-modified asphalt and 20%HTPUABDO at different temperatures was less than 6 min, and their Δ*η* values were close and relatively small, indicating uniform and stable distribution of the dispersed phase. In contrast, the introduction of DSDDP significantly increased Δ*η* of PUA, and the system required over 8 min to stabilize at different temperatures, reflecting its superior high-temperature flowability. The change is due to the breakage of some S-S covalent bonds at elevated temperatures, partially disrupting the original network structure. When external shear force stopped, part of the S-S bonds reorganized and crosslinked again, which accelerated the intermolecular exchange of PU and gradually formed a three-dimensional network structure in asphalt. The time-varying characteristics of PUA were manifested through dynamic exchange and topological rearrangement of S-S bonds. This is beneficial for enhancing workability within the construction range (>135 °C) and facilitating rapid performance recovery during service (40–60 °C). This rheological behavior is precisely the basis for PUA to achieve thermoresponsive self-healing. It is worth noting that the observed viscosity recovery is provisionally attributed to reversible structural recovery, but thermal equilibration and ordinary shear effects cannot be fully excluded. Repeated shear–rest cycles are needed to confirm reversible thixotropy.

### 3.3. Experimental Analysis of Healing Performance

#### 3.3.1. Fatigue-Healing Behavior

**Figure 8:**
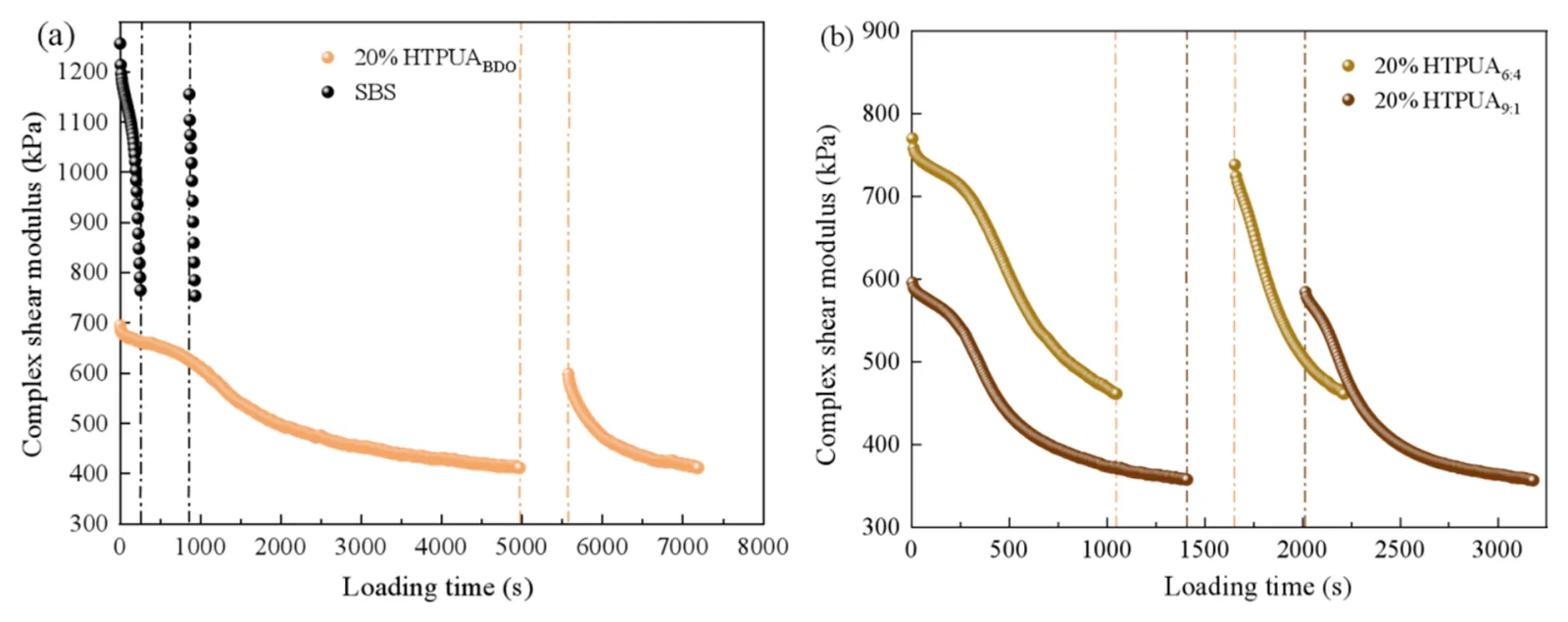
Fatigue–healing–fatigue curves of modified asphalt: (**a**) 20%HTPUA_BDO_ and SBS; (**b**) 20%HTPUA_6:4_ and 20%HTPUA_9:1._

To compare the effects of different S-S bond contents on the rheological performance recovery ability of PUA under fatigue conditions, the fatigue-healing behavior was examined. The initial loading temperature of the time-scanning test was set at 30 °C, with an interval of 40 °C and 10 min. The fatigue–healing–fatigue curves of different modified asphalts are shown in Figure 8.

**Figure 9:**
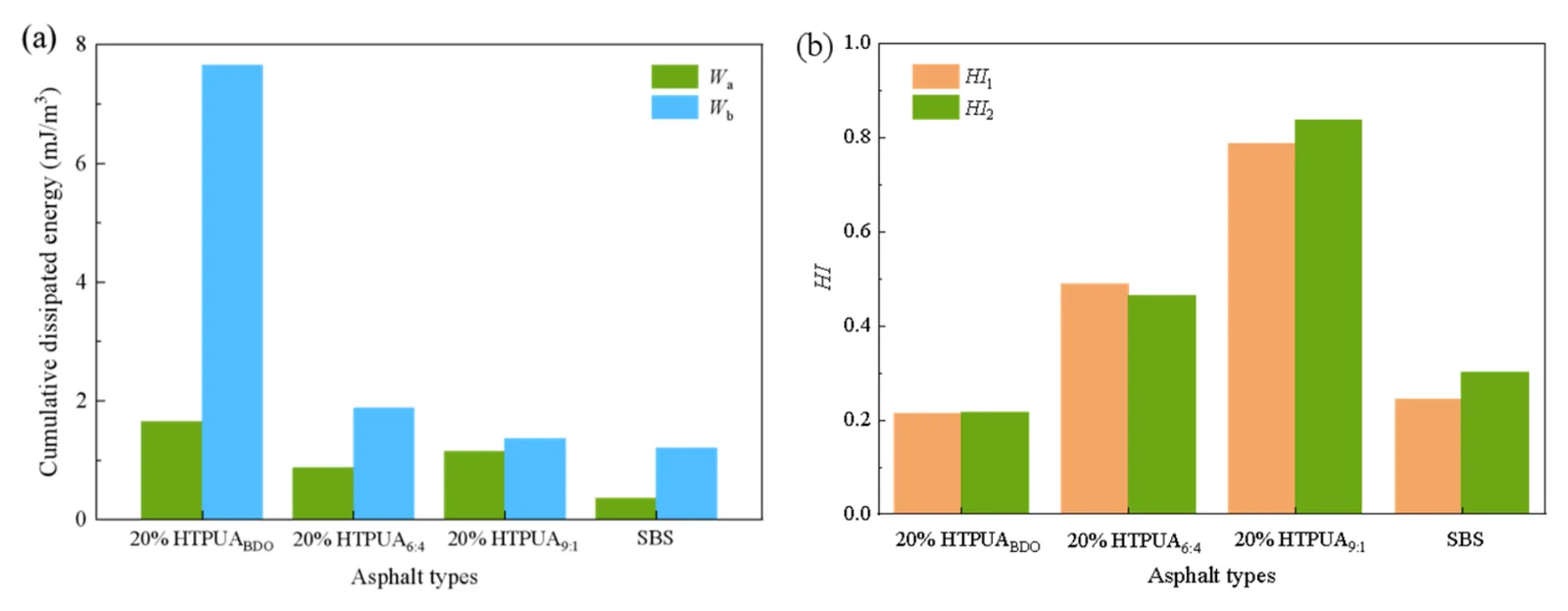
(**a**) Cumulative dissipated energy and (**b**) healing indexes of modified asphalt.

As illustrated in Figure 8, the $G_{0}^{*}$ of SBS-modified asphalt was the largest, but its complex modulus decreased rapidly with the test time. This is because its molecular chain might be partially relaxed under the shear effect when the dosage of SBS modifier was low, causing the elastic network structure to be prone to damage. Under the same intermittent conditions, the $G_{0}^{*}$ of PUA increased as the S-S bond content in the system rose, indicating an increase in the elastic components and energy storage modulus through crosslinking reactions. The stiffness of asphalt was enhanced, providing it with high deformation resistance and bearing capacity in the initial state. In addition, the introduction of S-S bonds significantly shortened the loading time of PUA to reach 40% damage degree before and after healing compared to 20%HTPUA_BDO_. This does not necessarily indicate lower absolute fatigue endurance or lower damage accumulation. PUA containing DSDDP had a higher initial complex modulus, and because part of the modulus decay arose from reversible S–S exchange and structural relaxation at medium temperature, the relative modulus threshold could be reached earlier even when the cumulative dissipated energy was lower. The *W*_a_ and *W*_b_ of modified asphalt calculated from the above curves are presented in Figure 9a, and *HI*_1_ and *HI*_2_ are shown in Figure 9b.

Figure 9a showed that the introduction of DSDDP significantly reduced the cumulative dissipation energy of PUA before and after healing, indicating a decrease in the cumulative dissipated energy under the selected loading protocol. It can be seen from the calculated *HI*_1_ and *HI*_2_ that the healing ability of 20%HTPUA_BDO_ was close to SBS-modified asphalt, while the healing indexes of PUA containing S-S bonds were significantly higher, confirming its thermoresponsive properties at medium temperatures. Taking 20%HTPUA_9:1_ for instance, its healing indexes during the given intermission were close to 0.8, with *HI*_1_ and *HI*_2_ being 3.88 and 3.67 times 20%HTPUA_BDO_, respectively. Thus, quantitative indexes demonstrated the promoting effect of increased S-S bond content on the fatigue-healing performance of PUA. This is attributed to the presence of van der Waals forces between the S-S bonds and amino groups in PU after bond breakage at medium temperatures, which reduced the microphase separation degree of PUA. In addition, the asymmetric alicyclic structure of the hard segment IPDI also provided sufficient chain mobility for the topological rearrangement of S-S bonds, thereby enabling PUA to maintain excellent mechanical properties. As the S-S bond content gradually increased, more free sulfur atoms generated from bond breakage participated in forming a dense crosslinked structure, which restricted intermolecular movement. The mobility of molecular chains deteriorates after damage, and the speed of rearrangement and reconnection slows down. Consequently, this impeded the rapid recovery of PUA to its original state after the removal of external forces.

#### 3.3.2. Prediction of Optimal Healing Temperature and Healing Time

The above experiments employed a fixed intermission. However, the dependence of asphalt on healing time and temperature led to varying repair effectiveness in different intermissions. A single intermittent condition could not reflect the healing behavior of asphalt under diverse environmental and load circumstances. To predict the optimal healing temperatures of PUA and SBS modified asphalt, the healing time was first set at 10 min during the intermission. Healing temperatures were then individually set at 30 °C, 40 °C, 50 °C, and 60 °C to simulate pavement temperatures under different climatic environments. The complex modulus variation curves of asphalt at different healing temperatures were obtained through fatigue–healing–fatigue tests, and their *HI*_1_ are calculated and shown in Table 2.

**Figure 10:**
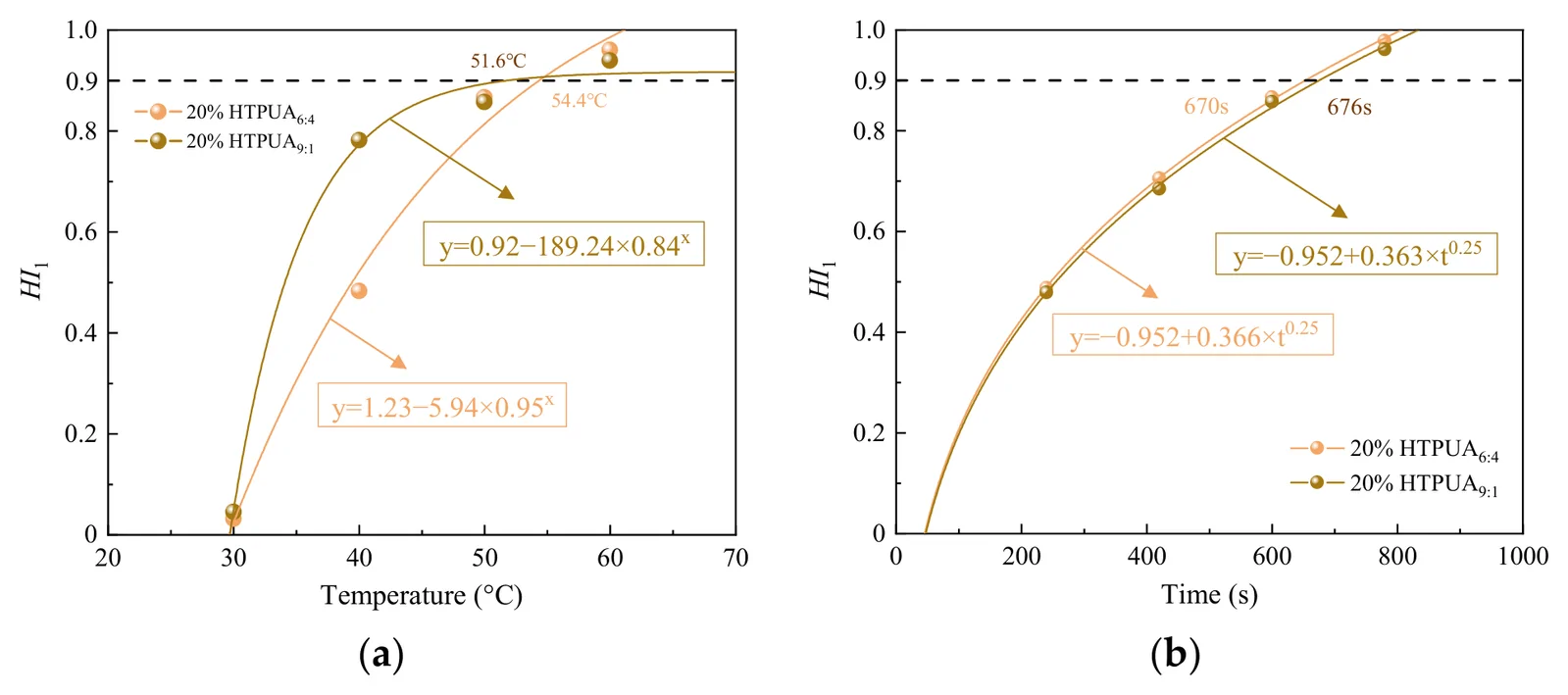
Curves of *HI*_1_ with (**a**) healing temperature and (**b**) healing time.

As illustrated in Table 2, the *HI*_1_ of different modified asphalt increased with the rising intermittent temperature. The *HI*_1_ of PUA containing S-S bonds was significantly higher than SBS modified asphalt at the same temperature. For the 2 types of thermoresponsive PUA, there was no obvious healing behavior at room temperature (around 30 °C). However, as the temperature rose above 40 °C, their healing capacity increased substantially, with *HI*_1_ exceeding 0.85 at 60 °C. This is because at higher temperatures, the viscosity of PUA decreased, its fluidity improved, and the molecular motion became more active, facilitating the rearrangement and interaction of molecules under fatigued states. Compared with 20%HTPUA_9:1_, 20%HTPUA_6:4_ with a higher S-S bond content exhibited lower *HI*_1_ within 40 °C. However, when the temperature rose to 50 °C and above, its healing index significantly increased and exceeded 20%HTPUA_9:1_. This indicated that the higher content of S-S bonds in 20%HTPUA_6:4_ was activated at medium to high temperatures and underwent more dynamic exchange interactions, accelerating molecular diffusion and segment reconnection through free flowing at the cracks. This also reflected the improved elasticity and flexibility of PU molecules, enabling the modified asphalt to recover to its original state more effectively under stress and maintain viscoelasticity within a broader temperature range. A nonlinear fitting of *HI*_1_ and healing temperature for 2 types of thermoresponsive PUA was performed according to the exponential function y = a – b × c^x^, as illustrated in Figure 10a.

As can be seen from the curves in Figure 10a, there was a favorable exponential relationship between *HI*_1_ and healing temperature (R^2^ > 0.95). *HI*_1_ exhibited the growth trend of accelerating initially and then slowing down as temperature rose, demonstrating that molecular diffusion and motion at medium temperatures led to rapid interfacial wetting and improved healing rates. However, when the healing index became excessively high (exceeding 0.9), the further increase in healing temperature had a negligible effect on its enhancement due to the limited mobility of molecular chains and increased energy dissipation of the system. Therefore, the temperature corresponding to *HI*_1_ = 0.9 was defined as the optimal healing temperature of asphalt (*T*_0_). It is worth noting that the temperature and time corresponding to *HI*_1_ = 0.9 are defined as threshold healing conditions, not necessarily the optimal practical healing conditions. It is concluded that *T*_0_ of the two PUA was below 55 °C. An increase in S-S bond content resulted in a higher *T*_0_ of PUA, which is related to the crosslinked structure with high thermal stability and large activation energy formed in the asphalt. Intermolecular contact, diffusion, and rearrangement were restricted, and molecular chain mobility within the microcrystalline regions was impeded, thereby reducing the healing reaction efficiency of PUA.

To further analyze the effect of intermittent time on the healing performance of thermoresponsive PUA and predict its optimal healing time, it is necessary to establish the healing behavior equations. According to diffusion theory, the healing capacity of asphalt mainly comprises the transient healing strength (*HI*_0_(T)) induced by cohesive forces at the interface and long-term healing strength (*HI*_h_(T,t)) caused by molecular diffusion [65]. Therefore, it can be expressed by the healing equation, as shown in Equation (8). *HI*(T, t) characterizes the cohesive strength formed by asphalt molecular diffusion, which varies with temperature (*T*) and is linearly correlated with the exponential of time (*t*), and thus can be expressed by Equation (9). The negative intercept is an extrapolation artifact of the empirical power-law model near zero time and does not imply negative instantaneous healing. Physically, *HI* ≥ 0. The model is valid only for t > 0. Considering the simplicity of the complex modulus ratio calculation method, which does not require the examination of dissipated energy within each loading cycle, *HI*_1_ demonstrates better applicability in the healing equation.

*HI*(T, t) = *HI*_0_(T) + *HI*_h_(T,t) (8)

*HI*(T, t) = *HI*_0_(T) + *K*(T)·*t*^0.25^ (9)

where *HI*(T, t) is the healing index of a certain intermission; *K*(T) is the healing rate.

Considering that the optimal healing temperature of thermoresponsive PUA was around 50 °C, an initial test temperature of 30 °C was set, with a fixed healing temperature of 50 °C during the intermission. Healing times of 4 min, 7 min, 10 min, and 13 min were respectively configured to simulate different load intermissions. The *HI*_1_ variation trend of PUA at different healing times was calculated through healing tests, and the results are presented in Figure 10b. The healing behavior equation for thermoresponsive PUA was obtained through power function fitting. Similarly, the *t* corresponding to *HI*_1_ = 0.9 was defined as the optimal healing time of asphalt (*t*_0_).

As presented in Figure 10b, at medium intermittent temperatures, the *HI*_1_ of different thermoresponsive PUA increased exponentially with healing time, exhibiting excellent correlation (R^2^ above 0.95). This indicated that the healing equation effectively described their healing behavior. As the intermittent time increased, the growth rate of the healing index gradually slowed, which is similar to the trend in Figure 10a. Longer intermittent time led to more rearrangement and crosslinking of PU molecular chains, which enabled favorable viscoelastic flow and intermolecular adhesion of PUA.

The fitting parameter *K* of the healing curve reflected the strength recovery rate of asphalt. As the S-S bond content increased, the *K* of the thermoresponsive PUA rose from 0.363 to 0.366, indicating that more PU molecular chains containing S-S bonds participated in the dynamic exchange reaction, thereby promoting performance recovery under fatigue states. All the *HI*_0_ values in the fitting curve were negative, indicating that under high loading conditions, asphalt under fatigue state was difficult to recover through transient healing strength. Within the intermittent time range of 600 s to 800 s, the healing index of PUA remained near 0.9. Moreover, the optimal healing times of 20%HTPUA_6:4_ and 20%HTPUA_9:1_ were calculated to be 670 s and 676 s, respectively, indicating that thermoresponsive PUA exhibited excellent self-healing potential under high temperature conditions. The fitted times are model predictions rather than directly measured values. It can be concluded that controlling the content of S-S bonds appropriately facilitated synergistic healing with the base asphalt. The crosslinking degree of the PU network structure formed on the asphalt surface was reduced, promoting molecular chain migration towards lower concentration areas, and consequently shortening the healing time of PUA.

#### 3.3.3. Adhesion-Healing Property

**Figure 11:**
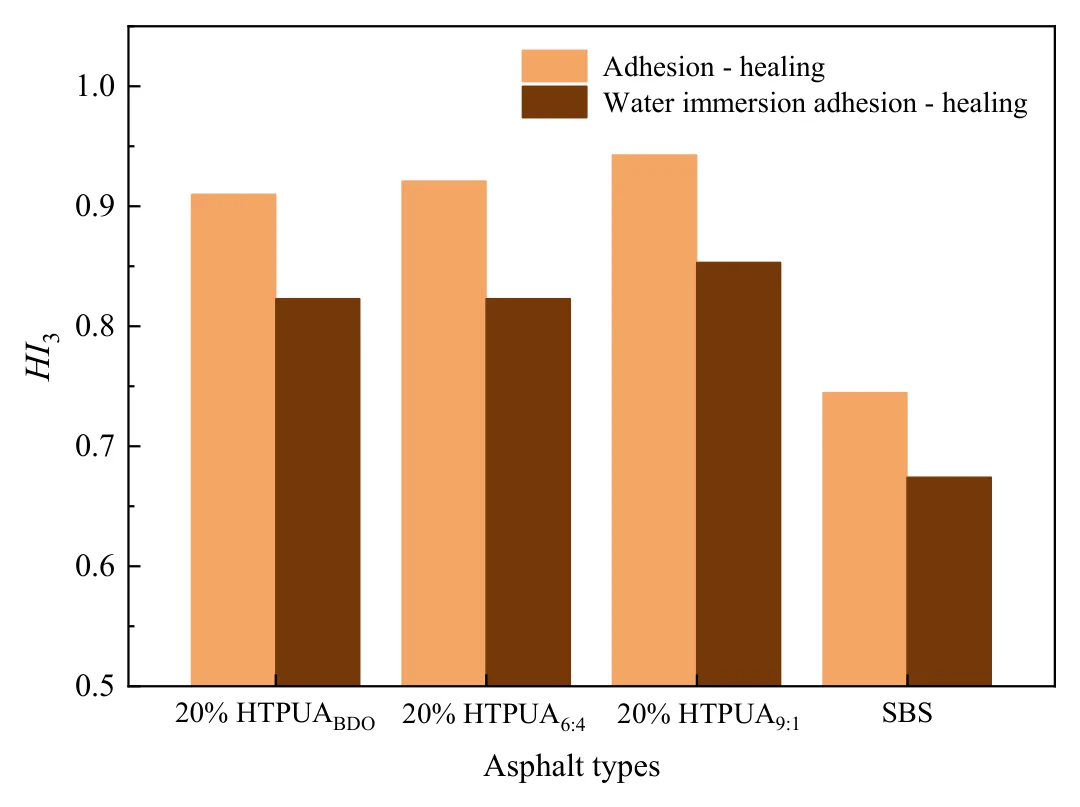
*HI*_3_ of modified asphalt.

Table 3 presents the *POTSi*_initial_, *POTS*_healing_ and failure modes of different modified asphalt. Failure modes are classified as adhesive failure (A) at the limestone surface, cohesive failure (C) within the binder, and mixed failure (M). The adhesion-healing and water immersion adhesion-healing properties of PUA and SBS modified asphalt calculated through the adhesion–healing–adhesion test are shown in Figure 11.

As illustrated in Figure 11, the adhesion-healing index *HI*_3_ of PUA consistently exceeded 0.9, which was significantly higher than SBS modified asphalt. PUA with HTPB as the soft segment exhibited great stability and flexibility at room temperature. Its adhesion to limestone was enhanced by forming hydrogen bonds during the healing process and contacting the polar group molecules on the aggregate surface. Compared with 20%HTPUA_BDO_, the *HI*_3_ of 20%HTPUA_6:4_ and 20%HTPUA_9:1_ containing S-S bonds increased by 1.1% and 3.3%, respectively. This indicated that the introduction of S-S bonds facilitated more molecular exchange on the asphalt surface after the BBS test, promoting molecular migration and thereby contributing to the reconstruction of PUA molecular bonding strength. Thus, the constructed active self-healing PUA system significantly enhanced adhesion and healing capacity at the asphalt-aggregate interface under long-term environmental effects. When shallow cracks developed in the thermoresponsive PUA during service, uniform heating or ultraviolet radiation could promote active healing of microcracks.

After water immersion healing, the *HI*_3_ of different modified asphalt all decreased, indicating that adhesion failure was more likely to occur under water conditions. This is because hydrophilic aggregates were more prone to contact with water in an immersion environment, causing partial stripping of the surface asphalt film and thereby weakening the bond strength between asphalt and aggregates. The addition of S-S bonds increased the *HI*_3_ of thermoresponsive PUA by 3% after immersion, demonstrating stronger water immersion adhesion. This is because the S-S bonds exhibited great hydrolysis resistance under neutral or weakly acidic conditions. Furthermore, as a hydrophobic material, PUA formed a stable crosslinked network between PU molecules and through S-S bonds. This alleviated the stripping effect of water molecules, so as to improve the water sensitivity of modified asphalt. It can be observed that thermoresponsive PUA reduced aggregate surface spalling and damage under both wet and dry conditions, thereby extending the pavement service life.

#### 3.3.4. Structure–Property Correlation Between HI and Flowability Parameters

**Figure 12:**
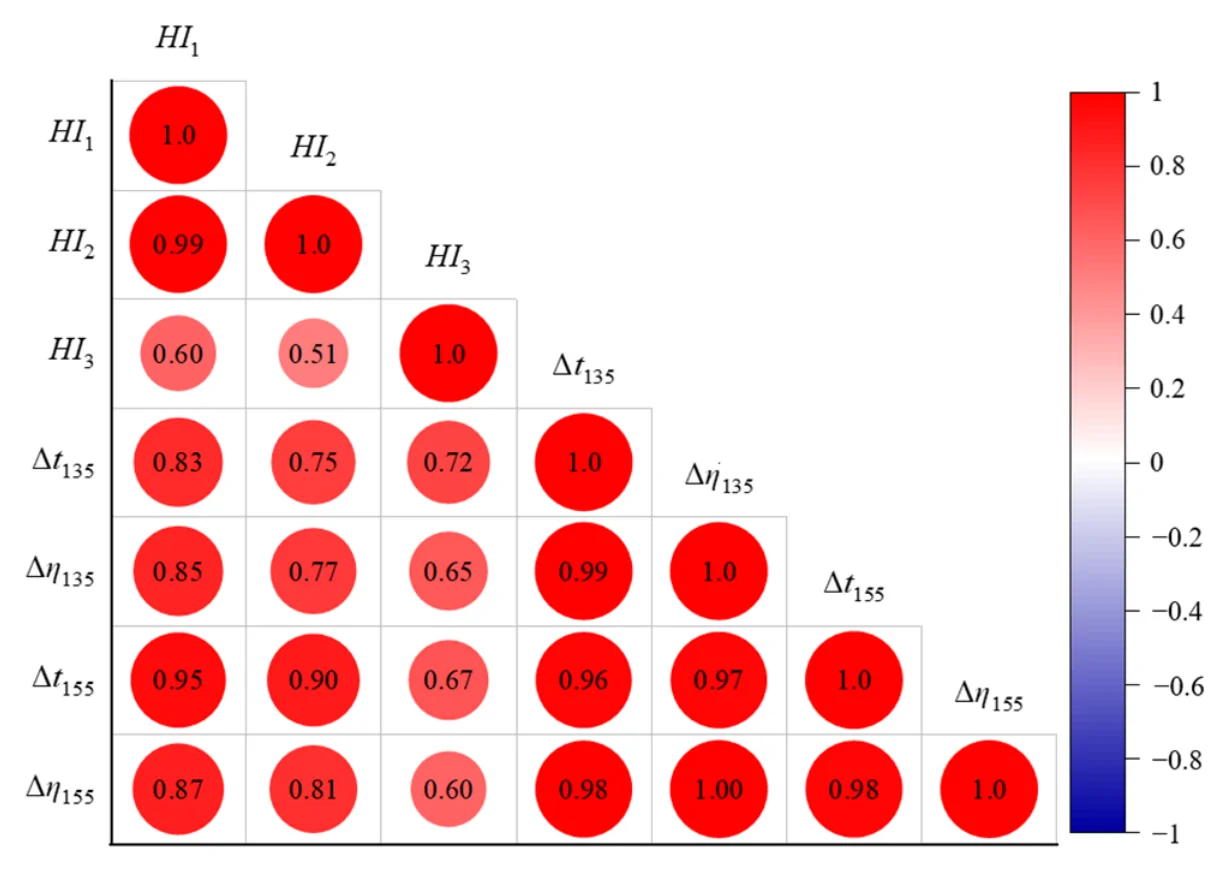
Correlation heat map between different healing indexes and flowability parameters.

Different indexes obtained from laboratory tests for evaluating the asphalt healing capacity reflect structural changes caused by internal fatigue damage. These indexes dynamically interact with external parameters such as healing temperature and time, affecting the rate of its structural reorganization. To investigate the structure–property relationship between the high-temperature flowability of asphalt and healing indexes derived from adhesion capacity, rheological response and energy dissipation, correlation heat map analysis was conducted on the healing indexes *HI*_1_, *HI*_2_ and *HI*_3_ of different PUA- and SBS-modified asphalt, as well as the flowability parameters Δ*t* and Δ*η* at the construction temperature range (135 °C and 155 °C). The results are presented in Figure 12. The heat map is based on *n* = 4 binder formulations; each value is the mean of three replicates. The correlation coefficients are reported as exploratory indicators.

Figure 12 demonstrated that *HI*_1_ and *HI*_2_ obtained from the time-scanning test exhibited identical trends, with a strong linear correlation (r = 0.99), indicating that both indexes reflected the damage and mechanical property changes in asphalt during loading. The shear resistance of asphalt partially corresponds to its fatigue resistance and damage accumulation under repeated loads. A faster decline in complex modulus typically indicates higher cumulative dissipated energy, where *HI*_2_ tends to express the energy loss, while *HI*_1_ emphasizes changes in stiffness and viscoelastic properties of the material. However, this relationship may be influenced by multiple factors, including loading conditions, temperature, structural composition and the initial state of modified asphalt. Moreover, the dynamic exchange of S-S bonds affects the cohesive force and viscoelastic deformation capacity of PUA.

In addition, it can be observed that the correlation between Δ*t* and Δ*η* at different temperatures was extremely high (r > 0.96), indicating excellent consistency of the two parameters in evaluating the high-temperature flowability of asphalt. The flowability parameters exhibited great correlation with the healing indexes *HI*_1_ and *HI*_2_ based on rheological response and energy dissipation, with r above 0.75. This suggested that the improved flowability contributed to the active recovery performance of asphalt after fatigue. S-S bonds can improve the high-temperature workability of PUA through continuous dynamic exchange and also accelerate molecular wetting, diffusion, and migration at microcracks. In contrast, the correlation between flowability parameters and *HI*_3_ was relatively weak, with r remaining between 0.6 and 0.7. This revealed that high plasticity might also cause asphalt softening and flowing excessively, thereby reducing its bond strength at the aggregate interface. Consequently, the healing of thermoresponsive PUA is the result of its inherent viscoelastic behavior and molecular motion at the aggregate interface, and it requires appropriate temperatures and sufficient time for the molecular chains to arrange and combine under external forces. Therefore, the S-S bond content needs to be adjusted according to actual engineering requirements. By balancing the flowability and bonding capacity of PUA, ideal active healing effects can be achieved.

### 3.4. Simulation of Healing Behavior

#### 3.4.1. Healing Process Analysis

**Figure 13:**
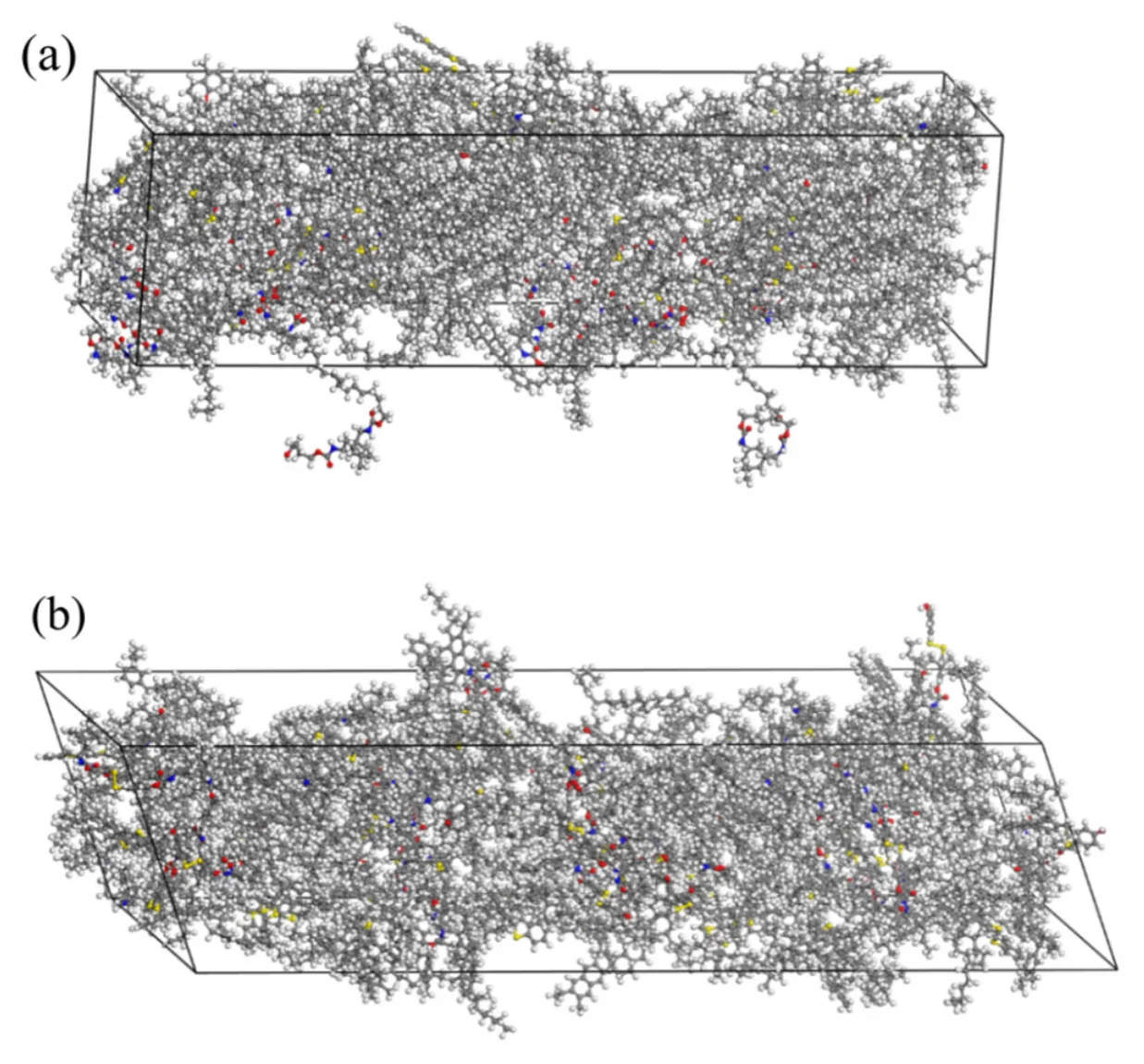
(**a**) HTPUA_BDO_/10 Å and (**b**) HTPUA_DSDDP_/10 Å after healing at 25 °C.

**Figure 14:**
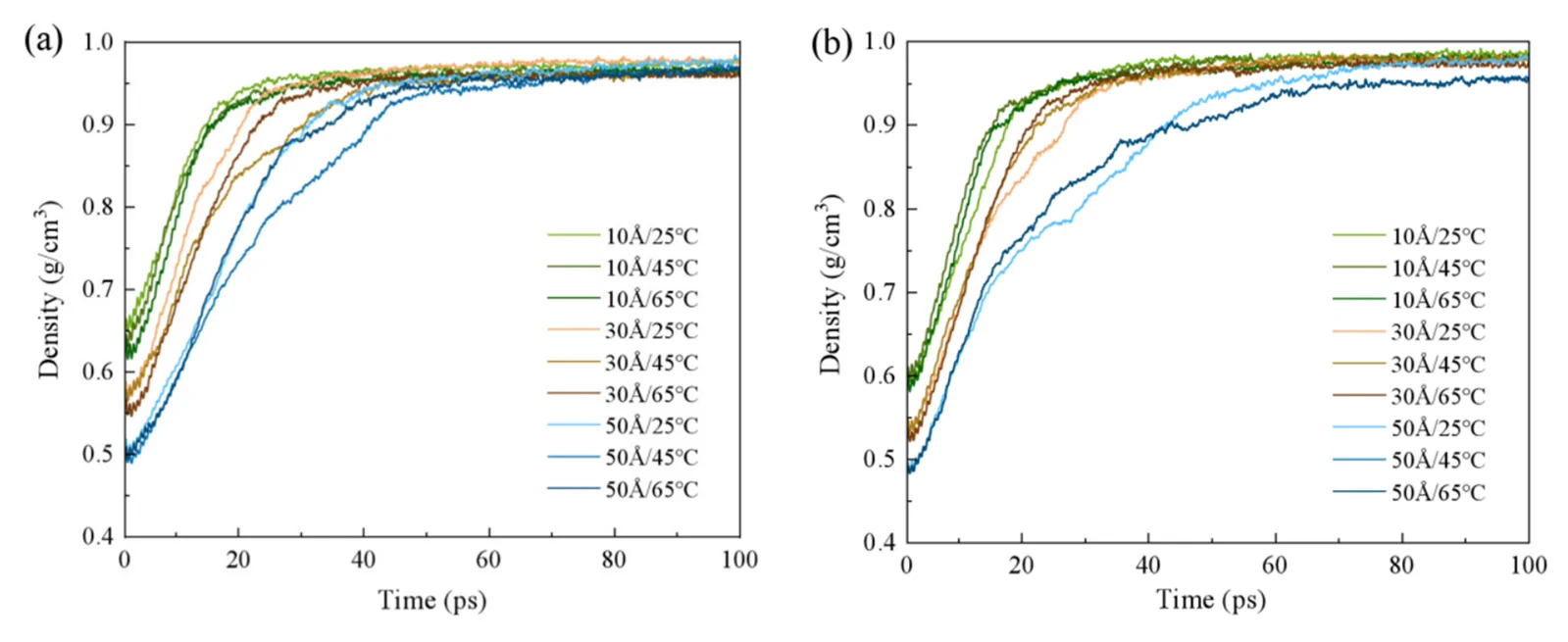
Density of (**a**) HTPUA_BDO_ and (**b**) HTPUA_DSDDP_ during healing at different temperatures.

Taking a spacing of 10 Å for instance, the PUA healing models after a 500 ps MD simulation at 25 °C are shown in Figure 13. It can be observed that PUA molecules approached and contacted each other, demonstrating significant diffusion behavior. To analyze the healing process of different types of PUA more effectively, the density variation with simulation time during the MD simulation process of HTPUA_BDO_ and HTPUA_DSDDP_ at different crack widths was first tested. To make different curves easier to recognize, data from the first 100 ps of simulation results were selected, as shown in Figure 14.

As illustrated in Figure 14, the density of different PUA exhibited a trend of initially rising rapidly, then continuing to increase slowly within the range of 20 ps to 40 ps, and stabilizing gradually within the range of 60 ps to 80 ps. From 90 ps to the end of the simulation, the density of PUA remained basically unchanged. The final density exceeded 0.9 g/cm^3^, which was close to the actual density of asphalt. During the initial healing stage, asphalt molecules at opposite ends were distant from each other with weak intermolecular forces. Under the influence of the NPT ensemble, they approached rapidly, which corresponded to the surface wetting stage of cracks during actual utilization. The subsequent stage occurred when asphalt molecules were relatively close, significantly enhancing intermolecular forces. This promoted further reduction in the unit cell length and increase in density, reflecting intermolecular diffusion at the crack surface. Finally, when asphalt molecules achieved complete mutual contact, the density of the system remained essentially constant. This suggested that during the stable diffusion stage, the interface of asphalt molecules underwent random combination and arrangement. Consequently, it can be summarized that the 5 stages of asphalt molecular healing are surface recombination, approach, wetting, interfacial diffusion and structural rearrangement.

**Figure 15:**
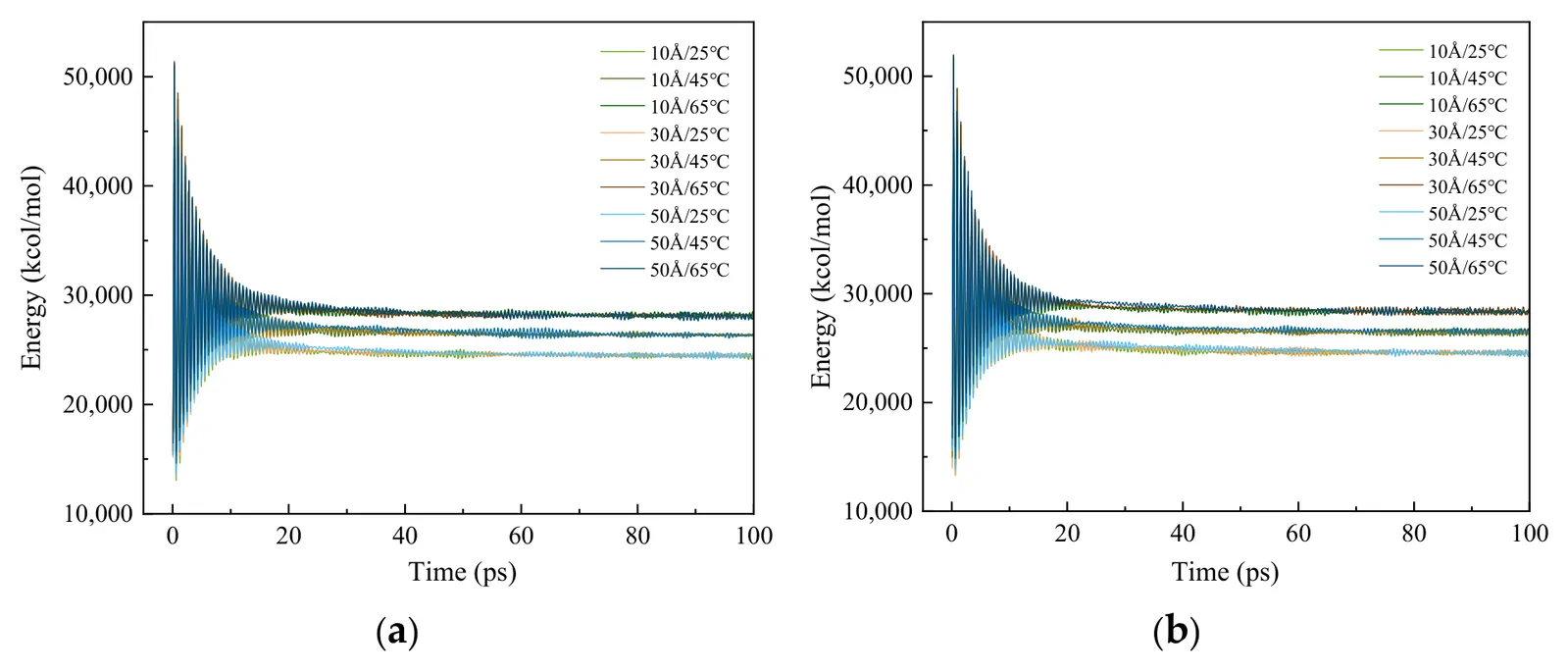
Energy of (**a**) HTPUA_BDO_ and (**b**) HTPUA_DSDDP_ during the healing process (0 ps~100 ps).

It can be observed through comparison that the density growth rate of different PUA molecular systems remained largely consistent within the first 20 ps. However, the density growth rate significantly decreased after 20 ps with the increase in initial spacing and the decrease in simulation temperature, resulting in the reduction in final density. Furthermore, the final density of HTPUA_DSDDP_ at the spacing of 10 Å and 30 Å exceeded 0.98 g/cm^3^, which was higher than HTPUA_BDO_. This demonstrated that the introduction of S-S bonds promoted the mutual filling and diffusion of PUA molecules, resulting in a more compact and stable system structure. Moreover, the simulation temperature also affects the kinetic energy and interaction of molecules. The total energy changes in different PUA models during the healing process were examined, and the results of the first 100 ps are presented in Figure 15.

As shown in Figure 15, the total energy of the PUA healing model exhibited initial intense fluctuations, then gradually decreased within the range of 30 ps to 50 ps, and finally stabilized after 60 ps till the end of the simulation. This indicated that the physical and thermodynamic properties of the system had essentially reached a state of partial equilibrium. The initial spacing had little effect on the final energy state of the PUA healing system, but the energy gradually increased with the rising of simulation temperature. The reason is that at higher temperatures, the molecular thermal motion and intermolecular interactions of PUA were enhanced, the average kinetic energy of the molecules increased, and the potential energy state changed accordingly, enabling the system to be in a state of high energy and entropy stability.

**Figure 16:**
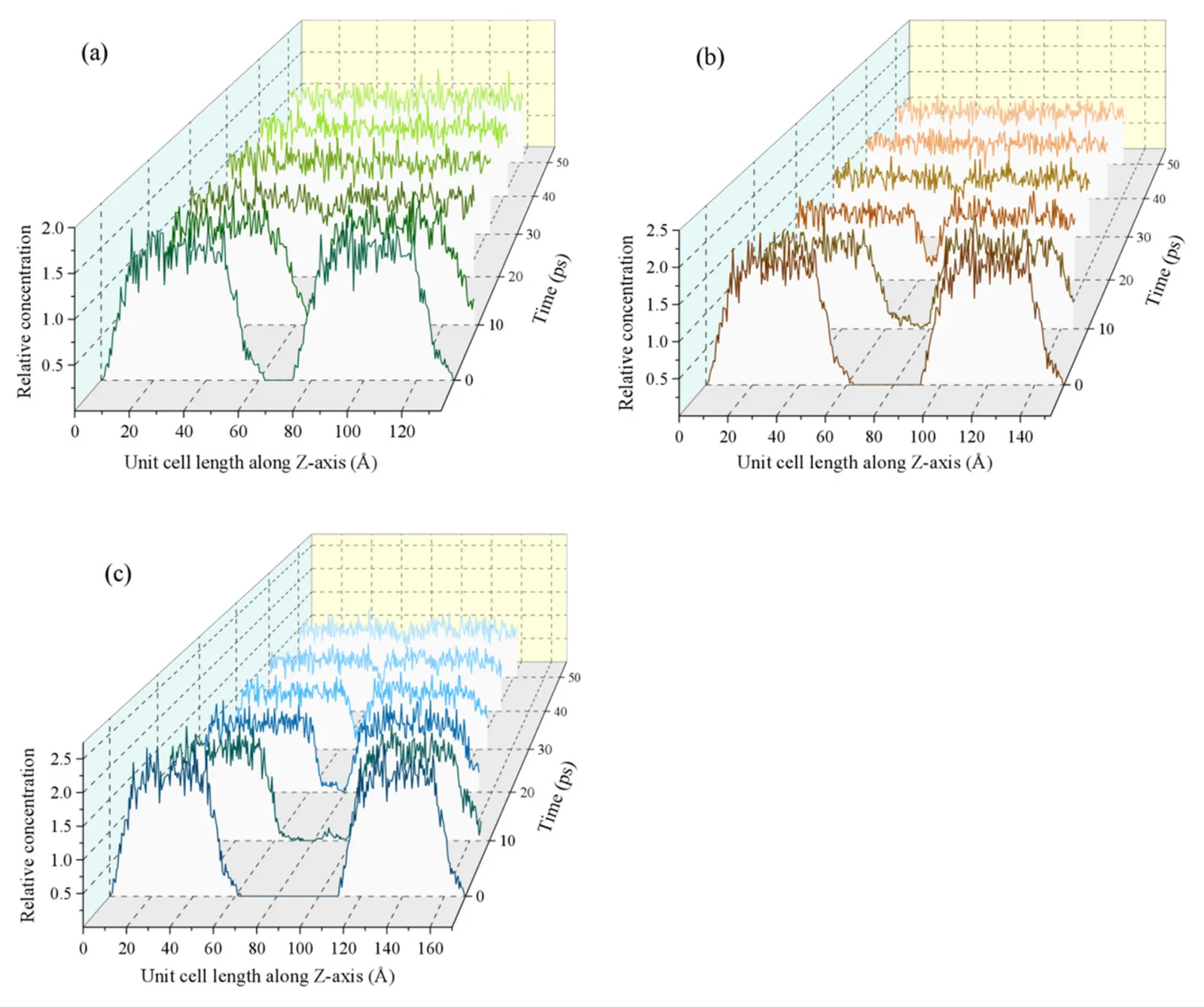
Relative concentration curves of HTPUA_DSDDP_ at (**a**) 10 Å, (**b**) 30 Å and (**c**) 50 Å.

The spatial distribution of molecules with simulation time during the healing process clearly indicates the healing stage. To this end, the relative concentration parameter for the material was introduced. The relative concentration (*C*_i_) of asphalt molecules at different times refers to the ratio of density at each spatial location to the average density of the system, which is calculated in Equation (10). When the material exhibits strong diffusion capacity at a certain location, its density approaches the average density of the system, and its relative concentration is approximately 1. Taking HTPUA_DSDDP_ at 25 °C as an example, the relative concentrations of its healing model with different spacings along the *Z*-axis were measured at simulation times of 0 ps, 10 ps, 20 ps, 30 ps, 40 ps, and 50 ps. Figure 16 illustrates its directional diffusion process.

(10) $$C_{i}=\frac{N_{i}}{V_{i}}\cdot \frac{V_{t}}{N_{t}}$$

where *N*_i_ and *V*_i_ are the number and volume of molecules at the selected position *i*, respectively; *N*_t_ and *V*_t_ are the total number of molecules and total volume in the system, respectively.

As shown in Figure 16, the center of the relative concentration curves before healing represents microcracks of different widths. PUA molecules on both sides were concentrated within a length range of 40 Å, with relative concentration peaks exceeding 2.0. During the initial simulation stage (0 ps to 20 ps), the concentration curves gradually shifted. A small number of asphalt molecules with extremely low concentration appeared in the central crack area, while the relative concentration of molecules on both sides decreased to between 1.5 and 2.0, indicating rapid movement of asphalt molecules and a healing trend. Subsequently, PUA molecules on both sides rapidly concentrated towards the central area between 20 ps and 40 ps, and the center of the curves gradually flattened. This indicated that molecules on both sides of the microcrack made contact and interacted. During the simulation time of 40 ps to 50 ps, the relative concentration curves approached stable straight lines, with the overall concentration values approaching 0.9 and peaks reaching 1.0, showing that healing was nearly complete and the PUA molecules had thoroughly diffused. Furthermore, the healing process decelerated as the initial spacing increased.

#### 3.4.2. Interfacial Stability and Cohesive Energy Density

The interaction energy at the asphalt interface reflects the compatibility and interfacial stability among its components. The non-bonding energy (*E_n_*), van der Waals energy (*E_v_*) and electrostatic energy (*E_ε_*) of the final conformation of the PUA molecular models were calculated with the method in Equation (11) to (13). The interaction energy of HTPUA_BDO_ and HTPUA_DSDDP_ at different simulation temperatures and crack widths is presented in Table 4, respectively. Higher interaction energy indicates stronger cohesive force and greater interfacial stability of asphalt, which helps to reduce the generation and propagation of cracks. Based on the MS Forcite decomposition energy output, it should be noted that *E_n_* consists of *E_v_*, *E_ε_* and *E_other_*. *E_other_* was not listed separately in Table 4, but can be obtained as *E_n_* − *E_v_* − *E_ε_*.

*E_n_* = *E_abn_* − (*E_an_* + *E_bn_*) (11)

*E_v_* = *E_abv_* − (*E_av_* + *E_bv_*) (12)

*E_ε_* = *E_abε_* − (*E_aε_* + *E_bε_*) (13)

where *a*, *b* and *ab* represent PU modifier, asphalt molecular, and PUA mixture system, respectively.

It can be seen from Table 4 that the *E_n_* values at the molecular interface of PUA were all negative, and the absolute values of *E_v_* were significantly greater than *E_ε_*, indicating that the van der Waals force became more dominant after healing. The absolute values of *E_n_* decreased with the rising temperature when the microcrack width was constant. This is because the thermal motion of asphalt molecules intensified and the kinetic energy increased, making the molecular arrangement more disordered and enabling them to overcome smaller intermolecular attractive forces for separation.

By comparing the interfacial interaction energy of molecular models under identical external conditions, it is evident that the *E_n_* values of HTPUA_DSDDP_ exceed −2000 kJ/mol, and the absolute values of each component of non-bonding energy were significantly higher than HTPUA_BDO_. This indicated that there was a strong intermolecular force between PUA-containing S-S bonds, which enhanced its cohesive strength. It was beneficial for the rapid movement and recombination of asphalt molecular chains at the interface of crack or damage, thereby significantly improving the healing efficiency. Hydrogen bonds and electrostatic energy contributed little to the healing behavior. The higher intermolecular interaction energy after healing completion restricted molecular movement, enhancing interfacial stability and maintaining structural integrity of asphalt.

**Figure 17:**
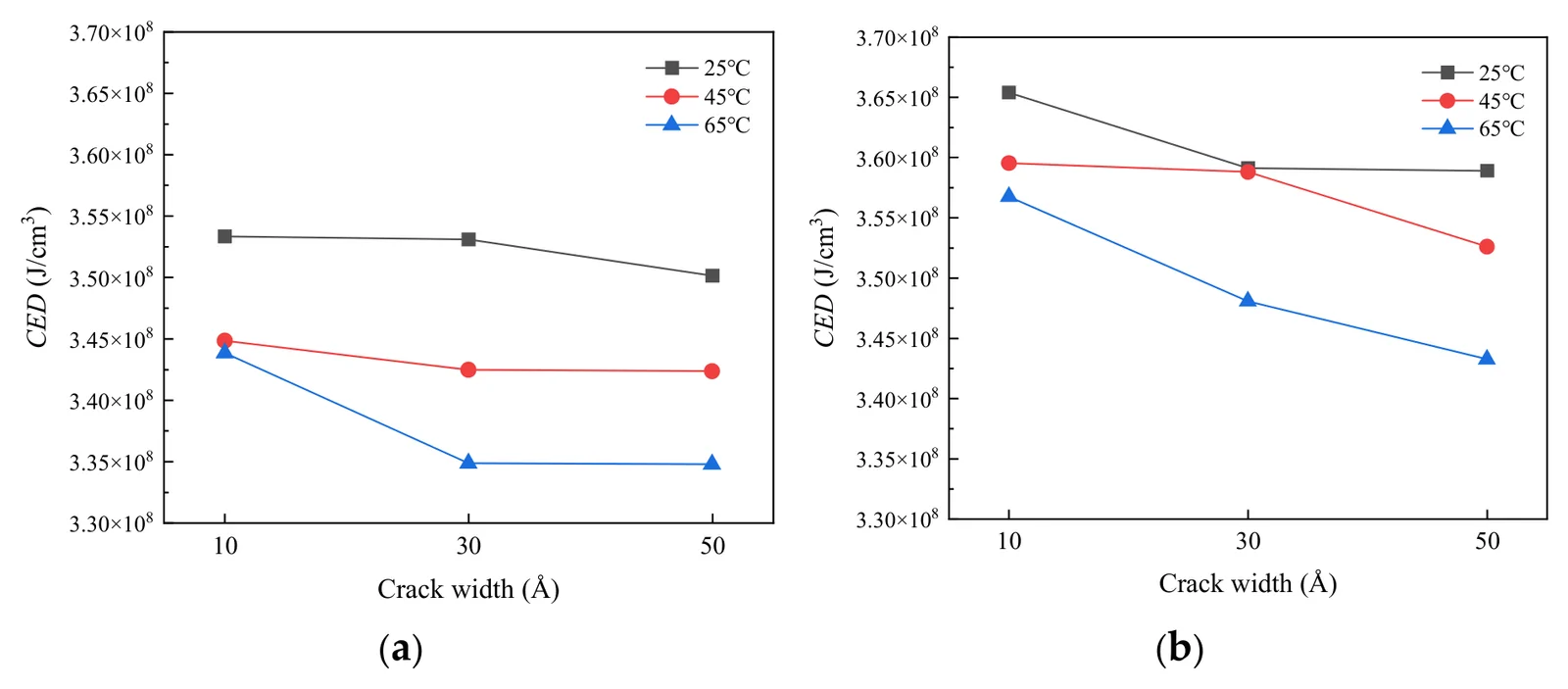
*CED* of (**a**) HTPUA_BDO_ and (**b**) HTPUA_DSDDP_.

Cohesive energy density (*CED*) characterizes the energy required for a unit volume of molecules to overcome internal molecular forces, manifesting as the energy of recombination at cracks during the healing process of PUA. *CED* reflects the healing efficiency of asphalt, where higher values indicate stronger motivation for intermolecular bonding during healing and faster crack closure rates. To this end, the *CED* of PUA after healing under different conditions was tested, with results presented in Figure 17.

As illustrated in Figure 17, the *CED* of PUA decreased considerably with the increasing crack width, indicating that greater initial damage progression hindered the healing process. The increase in simulation temperature led to a decline in *CED*, which is due to more intense molecular thermal motion, resulting in larger intermolecular distances and decreased interaction energy. Furthermore, HTPUA_DSDDP_ exhibited a significantly higher *CED* than HTPUA_BDO_ at a smaller initial spacing. This demonstrated that under the entanglement and crosslinking effects of PU molecules containing S-S bonds, HTPUA_DSDDP_ possessed greater polarity and intermolecular forces. Consequently, the asphalt structure became more stable, facilitating the rapid formation of new bonds after contact at the crack interface. In summary, the primary factors to affect the *CED* of PUA include temperature, molecular structure, and the type and strength of intermolecular forces.

#### 3.4.3. Diffusion Capacity During Healing Process

**Figure 18:**
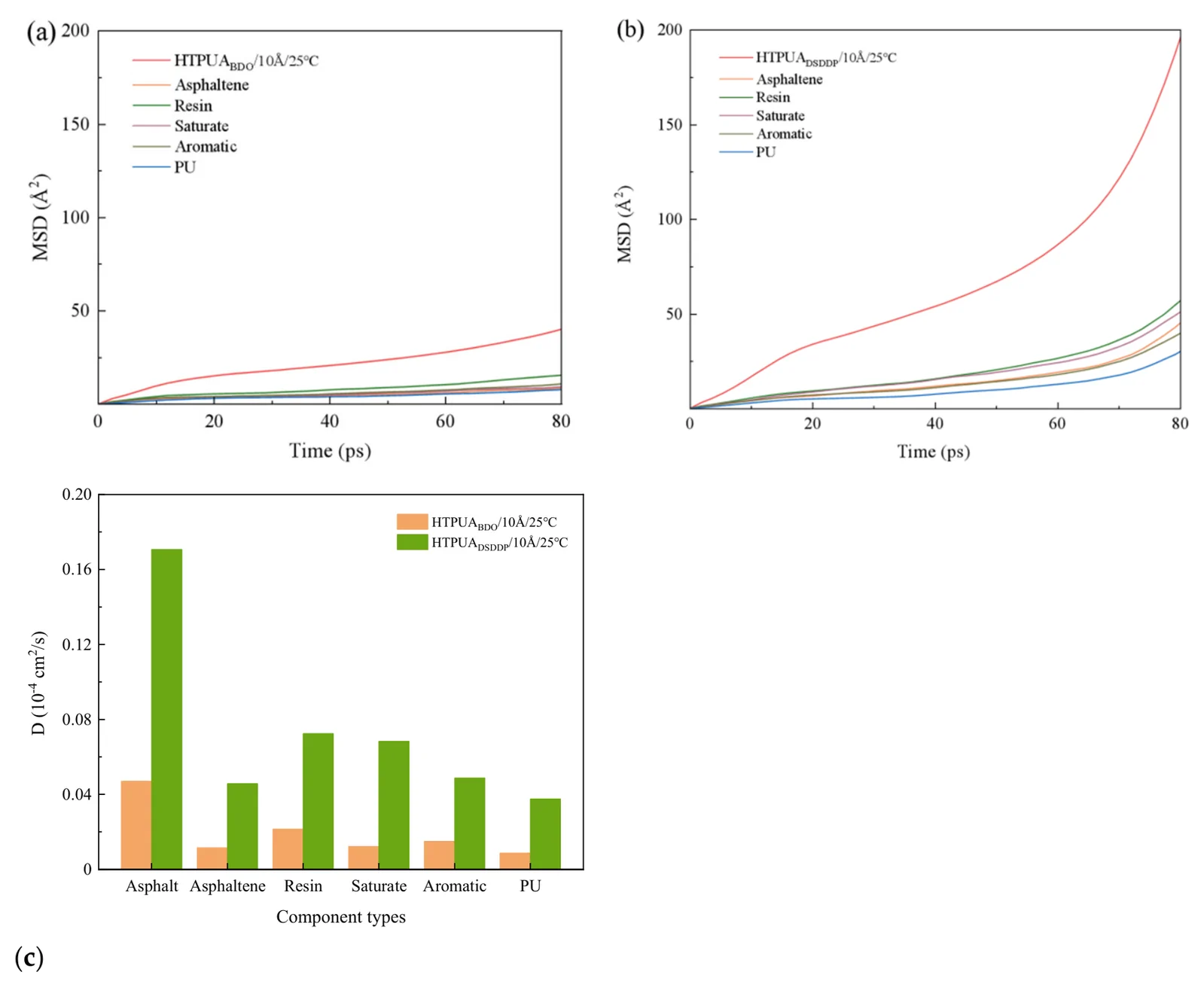
(**a**) HTPUA_BDO_ and (**b**) HTPUA_DSDDP_ MSD curves, and (**c**) *D* of PUA components.

The healing of asphalt at the molecular level primarily manifests as the random diffusion and aggregation of molecules from high concentration to low concentration positions until equilibrium is achieved. This diffusion motion can be regarded as a Markov process. The position of molecules changes at each time step during diffusion, and the probability of positional transition can be described by molecular thermal motion and collision. The center of mass for representative molecules of each component was identified through MS software and defined as a new set. The mean squared displacement (MSD) curves were employed to characterize the motion trends of various PUA components with time at different temperatures and crack widths. The diffusion coefficient (*D*), derived from the slope of the stable section of curves, was employed to characterize the average diffusion capability of the entire healing process and is calculated as in Equation (14). Higher *D* values indicate faster molecular diffusion rates. Differences in asphalt chemical composition and structure lead to variations in diffusion capacity among components during the healing process. Therefore, taking conditions of 10 Å and 25 °C as an example, MSD curves for different types of PUA models and their components were measured during the healing process (0 ps~80 ps). The results are shown in Figure 18a,b, with the corresponding calculated *D* presented in Figure 18c.

(14) $$D=\lim\frac{1}{6t}\mathrm{MSD}=\lim\frac{1}{6t}<{\left|r(t)\;-\;r(0)\right|}^{2}>$$

where *r*(*t*) is the displacement of particles at different times.

As shown in Figure 18a,b, the slopes of MSD curves for PUA and its components were relatively large in the initial stage (0 ps to 20 ps), then gradually decreased and exhibited a stable linear growth period. After 60 ps, the slope increased significantly again before stabilizing. This indicated that the healing of asphalt molecules was mainly divided into 3 different stages. Firstly, the PUA molecules on both sides rapidly migrated towards the crack center with a lower molecular concentration, primarily undergoing random diffusion. The second stage was contact diffusion, where molecules from opposite sides attracted each other, made contact, and were affected by intermolecular forces, leading to gradual crack healing. The final stage was stable diffusion, where accelerated thermal molecular motion promoted strength reconstruction at the crack site.

Taking HTPUA_BDO_/10 Å/25 °C for instance, the diffusion rates of its saturate and aromatic components were relatively fast, with *D* values of 1.23 × 10^−6^ cm^2^/s and 0.15 × 10^−6^ cm^2^/s, respectively. In contrast, PU molecules with long-chain structure exhibited the slowest diffusion, with *D* = 0.88 × 10^−6^ cm^2^/s. The variation in *D* is attributed to the smaller molecular weight and volume of light components, which had weaker intermolecular interaction. This facilitated easier motion and diffusion by overcoming intermolecular forces. Asphaltene and PU molecules possessed larger molecular weights, along with stronger van der Waals forces and hydrogen bonding interaction, which restricted their mobility. Furthermore, by comparing *D* values, it is evident that HTPUA_DSDDP_ exhibited significantly higher healing and diffusion capacities than HTPUA_BDO_. This indicated that during the healing process, HTPUA_DSDDP_ accelerated diffusion between different PUA molecules through S-S bonds, which facilitated the formation of a tighter structure as the system gradually stabilized, thereby enhancing its stability.

**Figure 19:**
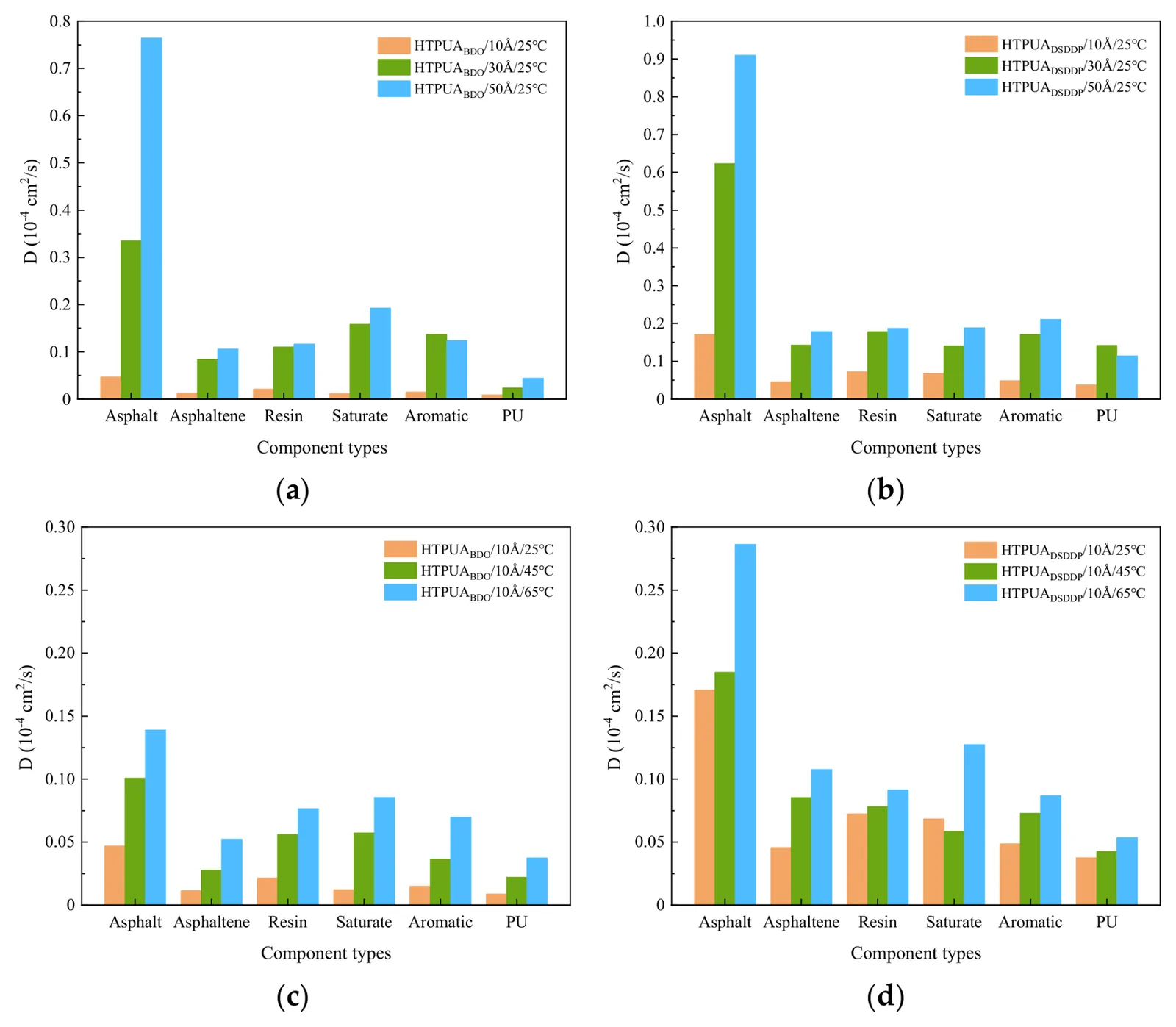
*D* of PUA components at different spacings (**a**,**b**) and temperatures (**c**,**d**).

The crack width determines the length of the molecular diffusion path. To investigate the effect of initial spacing on healing and diffusion of PUA and its components, MSD curves at spacings of 10 Å, 30 Å and 50 Å at 25 °C were tested, respectively, and the corresponding *D* values were calculated. The results are shown in Figure 19a,b.

As illustrated in Figure 19a,b, as the initial spacing gradually increased from 10 Å to 50 Å, the *D* of PUA molecules and their components showed a significant increase. This is because narrow cracks enhanced the intermolecular forces of asphalt, and reduced the motion distance and rate, which might impede free molecular movement. Comparative analysis revealed that the diffusion capacity of PUA components in different spacings maintained the order of light components > heavy components > PU. Moreover, the migration ability of components in HTPUA_DSDDP_ was significantly stronger than HTPUA_BDO_, reflecting stronger intermolecular interaction within it. Thus, appropriate spacing is conducive to providing sufficient healing space and ensuring effective intermolecular interaction. This suggests that the timing of pavement maintenance decisions significantly impacts treatment effectiveness. Timely maintenance during the middle stage of crack development or leveraging the inherent healing performance of pavement materials can help extend pavement service life.

To explore the effect of temperature changes on the diffusion behavior of PUA molecules and their components, MSD curves were measured at 25 °C, 45 °C, and 65 °C with a constant crack width of 10 Å. The corresponding *D* values were calculated and shown in Figure 19c,d.

Figure 19c,d indicated that at the same spacing, the diffusion capacity of PUA and its components increased with the rising of test temperature. This trend resulted from heightened molecular kinetic energy at elevated temperatures, leading to more active motion and weakened intermolecular forces. Consequently, asphalt molecules were more likely to overcome mutual adhesion and rearrange, manifesting as extensive bonding within cracks or damaged areas. Additionally, an increase in temperature above 25 °C reduced the viscosity of asphalt, which is conducive to the molecular flow and diffusion. Comparative analysis revealed that at the same temperature, the *D* of all components in HTPUA_DSDDP_ significantly exceeded HTPUA_BDO_. Furthermore, when ambient temperature rose from 45 °C to 65 °C, the *D* value of HTPUA_DSDDP_ increased substantially by 54.85%. This demonstrated that S-S bonds were activated above medium temperatures, thereby enhancing the healing efficiency of asphalt.

#### 3.4.4. Structure–Property Correlation Between HI and Simulation Indicators

**Figure 20:**
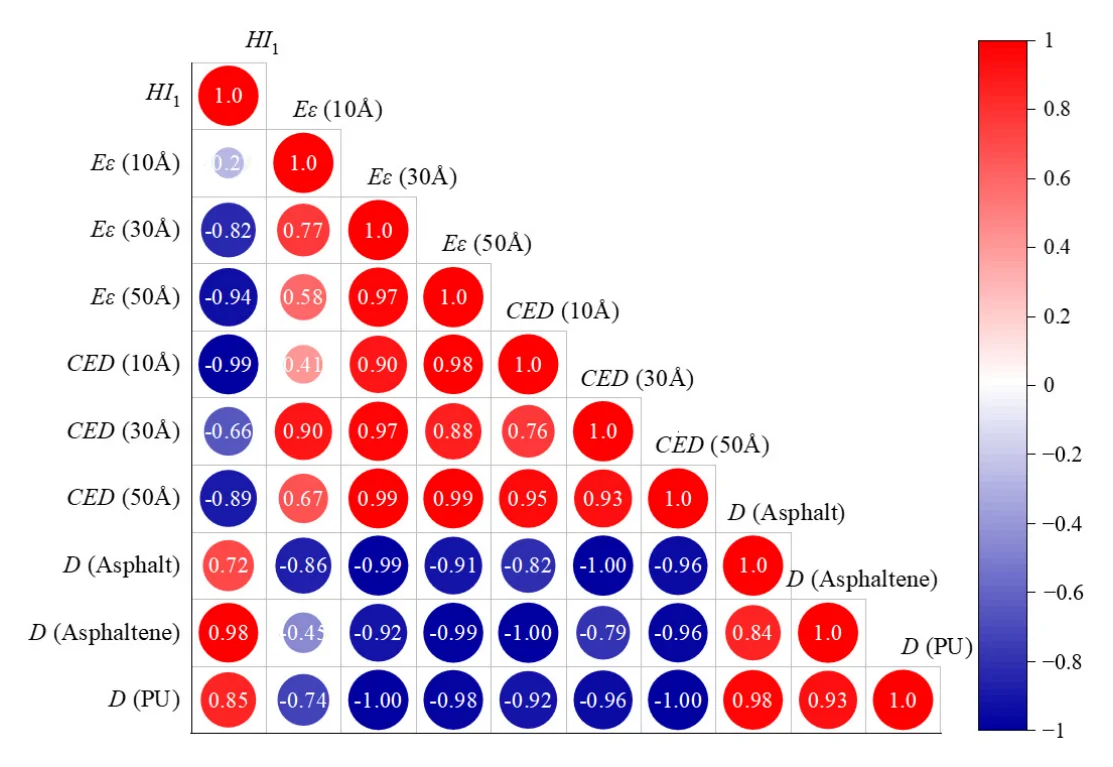
Correlation heat map between *HI*_1_ and different simulation indicators.

To explore the structure–property relationship between the macroscopic healing ability and molecular simulation indicators of thermoresponsive PUA within 20–60 °C, correlation analyses were conducted on the *HI*_1_, *E_ε_* and *CED* at different crack widths and *D* of different components of HTPUA_DSDDP_. The correlation heat map is shown in Figure 20. The heat map is based on *n* = 9 paired crack–width–temperature conditions. All paired *HI*_1_ values correspond to temperatures of 65 °C or below, which is within the medium-temperature range where dynamic S-S exchange is activated.

As illustrated in Figure 20, *HI*_1_ exhibited great correlations with various molecular simulation indicators (|r| > 0.7), with *D* of PUA components showing the strongest positive correlation (r > 0.85), indicating that the migration ability of the molecular chain is the main factor affecting healing efficiency. As the skeleton component of the asphalt system, polar groups in the macromolecular structure of asphaltene are more involved in the secondary formation of interfacial strength and, thus, exhibit the strongest correlation with healing indicators (r > 0.98). The relatively higher diffusion rate of PU molecular chains facilitates direct migration toward the crack interface, which is also a precondition for interfacial wetting, molecular entanglement and rearrangement of disulfide bonds. In contrast, both *E_ε_* and *CED* demonstrated negative correlations with *HI*_1_. This reflected that stronger intermolecular interactions help the rapid reorganization of the interfacial structure after crack propagation, serving as the thermodynamic driving force of the healing process. The above analysis indicated that molecular healing simulation indicators under different conditions possess predictive effects on the healing ability and long-term performance change in PUA under damage state, thereby providing guidance for optimizing the healing capacity of thermoresponsive PUA through molecular design.

## 4. Conclusions

In this study, polyurethane-modified asphalt (PUA) containing disulfide-based chain extenders was prepared to investigate its temperature-dependent flow and healing behavior. By combining macroscopic experiments and molecular simulations, the fatigue-healing and adhesion-healing properties were evaluated, and the healing behavior was simulated. Subsequently, structure–property relationships for the healing performance at different scales were assessed. The main conclusions obtained are as follows:

(1) The addition of chain extender DSDDP provided PUA with active response characteristics within the pavement temperature range of 40–60 °C, and slightly reduced its temperature sensitivity. The parameters Δt and Δη derived from the viscosity-time curve indicated that within the construction temperature range (above 135 °C), the topological rearrangement of S-S bonds facilitated the rapid recovery of high-temperature flowability for PUA in the short term.
(2) Appropriate content of S-S bonds promoted the active recovery of PUA and its contact with polar molecules on the aggregate surface through dynamic exchange, thereby significantly enhancing the fatigue-healing and adhesion-healing capacities of thermoresponsive PUA, with the healing index exceeding 0.8. The optimal healing temperature (T0) and healing time (t0) ranges for thermoresponsive PUA were defined as threshold healing conditions and were predicted to be 50–55 °C and 670~680 s through the healing equation, respectively. The results are consistent with the proposed dynamic S–S exchange mechanism.
(3) Molecular dynamics simulations indicated that the healing process primarily consisted of surface wetting, mutual contact, and stable diffusion stages. The diffusion rates of PUA components were accelerated with the increase in crack width and temperature, with lighter components diffusing faster than asphaltene and PU molecules. The intermolecular forces were weakened through dynamic exchanges of disulfide bonds, resulting in a denser structure of PUA after the healing completion.
(4) The multiscale structure–property relationship analysis indicated that the cohesive force of PUA and its bonding strength with aggregates jointly determine the healing capacity of modified asphalt mixtures under various environmental conditions. There is a strong structure–property relationship between the flowability and active healing capacity of PUA under fatigue conditions.

In conclusion, the thermoresponsive PUA based on the reversible disulfide bond system enables active and efficient performance adjustment within different temperature ranges. Investigations regarding long-life and sustainable pavements require further mixture-scale, aging, repeated-healing, and environmental-economic assessments.

## Figures and Tables

**Table 1:** Composition of PUA.

Asphalt Types	PU Content (%)	HTPB (mol)	IPDI (mol)	BDO (mol)	DSDDP (mol)
20%HTPUA_BDO_	20	0.02	0.06	0.04	0
20%HTPUA_6:4_	20	0.02	0.06	0.024	0.016
20%HTPUA_9:1_	20	0.02	0.06	0.036	0.004

**Table 2:** *HI*_1_ of modified asphalt at different healing temperatures.

Asphalt Types	30 °C	40 °C	50 °C	60 °C
20%HTPUA_BDO_	0.013	0.215	0.394	0.522
20%HTPUA_6:4_	0.031	0.483	0.867	0.961
20%HTPUA_9:1_	0.045	0.782	0.858	0.940
SBS	0.017	0.243	0.423	0.544

**Table 3:** Adhesion indicators of different modified asphalt.

Asphalt Types	*POTS* *i*_initial_	*POTS*_healing_	Failure Modes	*POTS*_healing_ (Immersion)	Failure Modes (Immersion)
20%HTPUA_BDO_	1.74	1.58	M	1.43	M
20%HTPUA_6:4_	2.30	2.12	M	1.89	M
20%HTPUA_9:1_	2.05	1.93	C	1.75	M
SBS	1.58	1.17	M	1.06	A

**Table 4:** Interaction energy of HTPUA_BDO_ and HTPUA_DSDDP_.

Asphalt Types	Crack Width (Å)	Temperature (°C)	*E_n_* (kJ/mol)	*E_v_* (kJ/mol)	*E_ε_* (kJ/mol)
HTPUA_BDO_	10	25	−1443.9	−1190.9	−213.8
		45	−1431.0	−1149.6	−241.9
		65	−1323.2	−1212.9	−71.4
	30	25	−1412.3	−1270.7	−101.9
		45	−1407.7	−1174.9	−193.8
		65	−1359.3	−1133.6	−186.9
	50	25	−1507.9	−1101.0	−367.9
		45	−1420.5	−1230.4	−150.6
		65	−1350.5	−1155.7	−155.6
HTPUA_DSDDP_	10	25	−2140.8	−1660.6	−433.4
		45	−2126.0	−1618.0	−461.6
		65	−2096.1	−1656.9	−392.6
	30	25	−2167.0	−1629.8	−491.0
		45	−2115.3	−1612.2	−456.7
		65	−2052.4	−1636.8	−369.5
	50	25	−2197.4	−1601.5	−549.7
		45	−2149.8	−1626.5	−478.2
		65	−2135.7	−1681.5	−407.9

## Data Availability

The original contributions presented in this study are included in the article. Further inquiries can be directed to the corresponding author.

## Supplementary Materials

The following supporting information can be downloaded at: https://www.mdpi.com/article/10.3390/polym18212711/s1. Table S1: Technical indexes of chain extenders; Table S2: Technical properties of HTPB and IPDI; Table S3: Technical indicators of asphalt; Table S4: Molecule numbers of base asphalt models.

## Abbreviations

The following abbreviations are used in this manuscript:

PUA	polyurethane-modified asphalt
FTIR	Fourier transform infrared spectroscopy
DSR	dynamic shear rheometer
POTS	pull-off tensile strength
MD	molecular dynamics
CED	cohesive energy density
MSD	mean squared displacement
