# Supplementary material for: Self-Healing Behavior of Medium-Temperature Thermoresponsive Polyurethane-Modified Asphalt: Multiscale Structure–Property Relationships
Source: Polymers (Basel). 2026 Nov 1;18(21):2711. doi: 10.3390/polym18212711 (PMC13766018; doi:10.3390/polym18212711)
Supplement: Supplementary file 1 [file polymers-18-02711-s001.zip › polymers-4613773-supplementary.pdf]

Supplementary File

1. Raw material parameters (Supplementary section 2.1 Materials and preparation process)

Table S1 Technical indexes of chain extenders.

Test properties	Unit	BDO	DSDDP
Appearance (25°C)	/	Colorless viscous oily liquid	White to yellowish solid powder
Melting point	°C	20.2	152
Boiling point	°C	228	437.9
Density	g/cm^3	1.02	1.44

Table S2 Technical properties of HTPB and IPDI.

Test properties	Unit	HTPB	IPDI
Appearance (25°C)	/	Yellowish transparent liquid	Transparent liquid
Molar mass	g/mol	2740	222
Hydroxyl value	mgKOH/g	71	/
NCO content	%	/	37.5-37.8
Hydrolyzed chlorine	ppm	/	<200
Viscosity	mPa·s	3200 (40°C)	15
Moisture content	%	0.022	/

Table S3 Technical indicators of asphalt.

Test properties	Unit	70$^{\#}$	Test method
Penetration (25°C)	0.1mm	73	T 0604
Softening point	°C	44.5	T 0606
Ductility (5°C)	cm	Fracture	T 0605
Density (15°C)	g/cm^3	1.031	T 0603
Dynamic viscosity (135°C)	Pa·s	0.69	T 0625

2. Construction process of polyurethane asphalt molecular models (Supplementary section 2.5 Construction of PUA molecular healing model)

(1) Repeatedly adjusting the number and ratio of each molecule were adjusted repeatedly to ensure that the component proportions in the constructed model were close to the results of SARA test. The molecular models of asphalt before and after aging were eventually established, and the specific parameters of the models are summarized in Table 4.

Table S4. Molecule numbers of base asphalt models.

Component	Number	Chemical formula	Molecular number	Proportion
Asphaltene	A	$C_{42}H_{54}O$	3	13.28
	B	$C_{66}H_{81}N$	2	
	C	$C_{51}H_{62}S$	2	
Resin	A	$C_{40}H_{59}N$	3	25.04
	B	$C_{40}H_{60}S$	3	
	C	$C_{18}H_{10}S_2$	11	
	D	$C_{36}H_{57}N$	3	
	E	$C_{29}H_{50}O$	3	
Saturate	A	$C_{30}H_{62}$	7	17.12
	B	$C_{35}H_{62}$	7	
Aromatic	A	$C_{30}H_{46}$	20	44.56
	B	$C_{35}H_{44}$	18	

(2) According to the synthesis reaction principles of PU, the PU modifier models with BDO and DSDDP as the chain extenders were separately established by the "Build Polymers" module of Materials Studio (MS) software. The ratio of diol, isocyanate and chain extender in the constructed PU molecular chains was 1:3:2. It is calculated that after inserting 4 PU molecular chains into the asphalt molecular model, the PU content was about 20% of the asphalt mass, which was close to the PUA prepared in laboratory tests.

(3) The molecular models of base asphalt and PU modifier were then assembled through the "Amorphous Cell Calculation" function of the MS software. The initial density of the constructed PUA model was 0.1 g/cm^3, and then the model was successively subjected to energy optimization and 40,000 steps of geometry optimization through the "Forcite" module, thereby minimizing the system energy.

(4) The model was then annealed for 5 cycles with a total of 150 ps under the canonical ensemble (NVT) to eliminate the local high energy sites. The model was finally subjected to a 500 ps of dynamic simulation at 25°C under the grand canonical

ensemble (NPT) with constant pressure and temperature, so that the molecules in the system were sufficiently mixed until stabilization.

(5) The force field adopted in the above process is COMPASS II, which is currently widely used to simulate the thermodynamic properties of various polymers. The sum approaches of electrostatic energy and van der Waals force were the Ewald and Atom based respectively, and the calculation accuracy was Fine.
